# Core Binding Factors are essential for ovulation, luteinization, and female fertility in mice

**DOI:** 10.1038/s41598-020-64257-0

**Published:** 2020-06-18

**Authors:** Somang Lee-Thacker, Hayce Jeon, Yohan Choi, Ichiro Taniuchi, Takeshi Takarada, Yukio Yoneda, CheMyong Ko, Misung Jo

**Affiliations:** 1 Department of Obstetrics and Gynecology, Chandler Medical Center, 800 Rose Street, University of Kentucky, Lexington, KY 40536-0298 USA; 2 Laboratory for Transcriptional Regulation, RIKEN Center for Integrative Medical Sciences 1-7-22, Suehiro-cho, Tsurumi-ku, Yokohama, Kanagawa 230-0045 Japan; 3 Department of Regenerative Science, Okayama University Graduate School of Medicine, Dentistry and Pharmaceutical Sciences, Okayama, 700-8558 Japan; 4 Section of Prophylactic Pharmacology, Kanazawa University, Venture Business Laboratory 402, Kakuma-machi, Kanazawa, Ishikawa 920-1192 Japan; 5 Department of Comparative Biosciences, College of Veterinary Medicine, 2001 South Lincoln Avenue, University of Illinois at Urbana-Champaign, Urbana, Illinois 61802 USA

**Keywords:** Transcriptional regulatory elements, Infertility, Reproductive disorders

## Abstract

Core Binding Factors (CBFs) are a small group of heterodimeric transcription factor complexes composed of DNA binding proteins, RUNXs, and a non-DNA binding protein, CBFB. The LH surge increases the expression of *Runx1* and *Runx2* in ovulatory follicles, while *Cbfb* is constitutively expressed. To investigate the physiological significance of CBFs, we generated a conditional mutant mouse model in which granulosa cell expression of *Runx2* and *Cbfb* was deleted by the *Esr2Cre*. Female *Cbfb*^*flox/flox*^*;Esr2*^*cre/+*^*;Runx2*^*flox/flox*^ mice were infertile; follicles developed to the preovulatory follicle stage but failed to ovulate. RNA-seq analysis of mutant mouse ovaries collected at 11 h post-hCG unveiled numerous CBFs-downstream genes that are associated with inflammation, matrix remodeling, wnt signaling, and steroid metabolism. Mutant mice also failed to develop corpora lutea, as evident by the lack of luteal marker gene expression, marked reduction of vascularization, and excessive apoptotic staining in unruptured poorly luteinized follicles, consistent with dramatic reduction of progesterone by 24 h after hCG administration. The present study provides *in vivo* evidence that CBFs act as essential transcriptional regulators of both ovulation and luteinization by regulating the expression of key genes that are involved in inflammation, matrix remodeling, cell differentiation, vascularization, and steroid metabolisms in mice.

## Introduction

Successful ovulation and subsequent development of the corpus luteum (CL) are fundamental for female fertility. The luteinizing hormone (LH) surge initiates these processes by activating responsive second messengers in preovulatory follicle cells. The activation of these second messenger systems leads to the induction and activation of transcriptional regulators that directly control the expression of a diverse array of genes encoding intra- and extra-cellular factors [reviewed in^1,2^]. These factors execute precisely coordinated actions to accomplish successful ovulation and subsequent development of the CL. Accumulating evidence using transgenic mouse models has identified such transcription factors including progesterone receptor (PGR), CCAAT/enhancer-binding protein alpha/beta (CEBPA/B), peroxisome proliferator-activated receptor γ (PPARG), the liver receptor homolog-1 (NR5A2), and nuclear receptor-interacting protein 1(NRIP1)^3–7^. Recent studies by our and other laboratories have shed light on a small family of transcription factors, the Core Binding Factors (CBFs), as critical mediators of the periovulatory process^8–10^.

The CBF is a heterodimeric transcription factor complex composed of DNA binding alpha subunits (RUNX1, RUNX2, and RUNX3) and a non-DNA binding beta subunit (CBFB). CBFs play fundamental roles in tissue development, with each RUNX protein playing a distinct role(s) in the development of various tissues^11–15^. As a partner for all RUNX proteins, CBFB has shown to enhance the DNA binding activity and stability of RUNX proteins, thus is instrumental in the overall activity of CBFs^16–18^.

In the ovary, the LH surge increases the expression of *Runx1* and *Runx2* in preovulatory follicles, while *Cbfb* is ubiquitously and constitutively expressed^9,19–21^. Functional redundancy among RUNX proteins has been reported in cancer cells as well as normal cells^22,23^. In the previous study using rat granulosa cells, we also showed that RUNX1 and RUNX2 have a redundant function in regulating the expression of *Hapln1*^24^. In addition, evidence from *in vivo* and *in vitro* studies showed that RUNX2 down-regulated *Runx1* expression in forming CL in rats and mice^10,25^, indicating a cross-regulation between different family members in ovarian cells. Moreover, mice deficient of the *Runx1*, *Runx2*, or *Cbfb* gene die at the embryonic stage or shortly after birth^12,13,26^. These findings present challenges in determining the role of each RUNX protein as well as the functional significance of overall CBFs in the adult ovary *in vivo*.

Recently, we generated granulosa cell-specific *Cbfb* knockout mice using two different *Cre* lines (*Cyp19*^*cre*^ and *Esr2*^*Cre/+*^)^9,10^. Our rationale was to reduce the activity of both RUNX1 and RUNX2 by deleting *Cbfb* expression in granulosa cells. Both transgenic mouse lines were subfertile^9,10^, with *Cbfb*^*flox/flox*^;*Esr2*^*Cre/+*^ mice displaying stronger phenotypical changes compared to those of *Cbfb*^*flox/flox*^*;Cyp19a1*^*cre*^ mice^9,10^. In agreement with these findings, *Cbfb*^*flox/flox*^;*Esr2*^*Cre/+*^ mice showed higher efficiency in deleting *Cbfb* expression in granulosa cells than *Cbfb*^*flox/flox*^*;Cyp19*^*cre*^ mice^9,10^. This might be in part due to the timing difference in *Esr2* and *Cyp19a1* expression in primary vs. secondary follicles, respectively, or incomplete removal of the gene by *Cyp19-Cre* throughout all follicles^27,28^. Despite severe subfertility, *Cbfb*^*flox/flox*^*;Esr2*^*Cre/+*^ mice showed only ~65% reduction in ovulation rates, and the majority of unruptured antral follicles transformed into morphologically normal-looking CL, although progesterone levels were reduced on day 3 post-hCG administration compared to those in wild-type animals^10^. Consistent with these changes, the expression of two ovulatory genes (*Edn2* and *Ptgs1*) and several luteal genes (*Lhcgr*, *Sfrp4*, *Wnt4*, *Ccrl2*, *Lipg*, *Saa3*, and *Ptgfr*) was compromised in these mutant mouse lines^9,10^. These data indicated that the deletion of *Cbfb* in granulosa cells resulted in a partial reduction of ovulation rate and CL function in mice.

In establishing granulosa cell-specific *Cbfb* knockout mice, we presumed that *Cbfb* deletion would result in the greatly diminished activity and degradation of RUNX proteins. For RUNX1 protein, this was true in ovulatory follicles^10^. However, the LH surge/hCG-induced expression of *Runx2* (mRNA and protein) was not affected in the ovary of *Cbfb*^*flox/flox*^*;Esr2*^*Cre/+*^ mice^10^, suggesting that RUNX2 is less susceptible to degradation in granulosa cells and may function even in the absence of CBFB. Therefore, it is important to determine whether the additional deletion of *Runx2* would result in more profound defects in ovulation and luteal development.

To address this question, we generated transgenic mice deficient of both the *Runx2* and *Cbfb* gene in granulosa cells (*Cbfb*^*flox/flox*^*;Esr2*^*Cre/+*^*;Runx2* ^*flox/flox*^). This mutant mouse was used to assess ovulation rate, CL development, gene expression, ovarian morphology, hormone profiles, and fertility. Herein, we demonstrated that the removal of RUNX1, RUNX2, and CBFB in periovulatory granulosa cells resulted in a complete blockage of ovulation and CL development in the mouse ovary, consequently causing infertility. This study also identified a number of additional genes that are potential downstream targets of RUNXs/CBFB, which are involved in the ovulatory process and luteinization in the mouse ovary.

## Results

### Generation and verification of granulosa cell-specific double deletion of *Cbfb* and *Runx2* in mice

**Figure 1:**
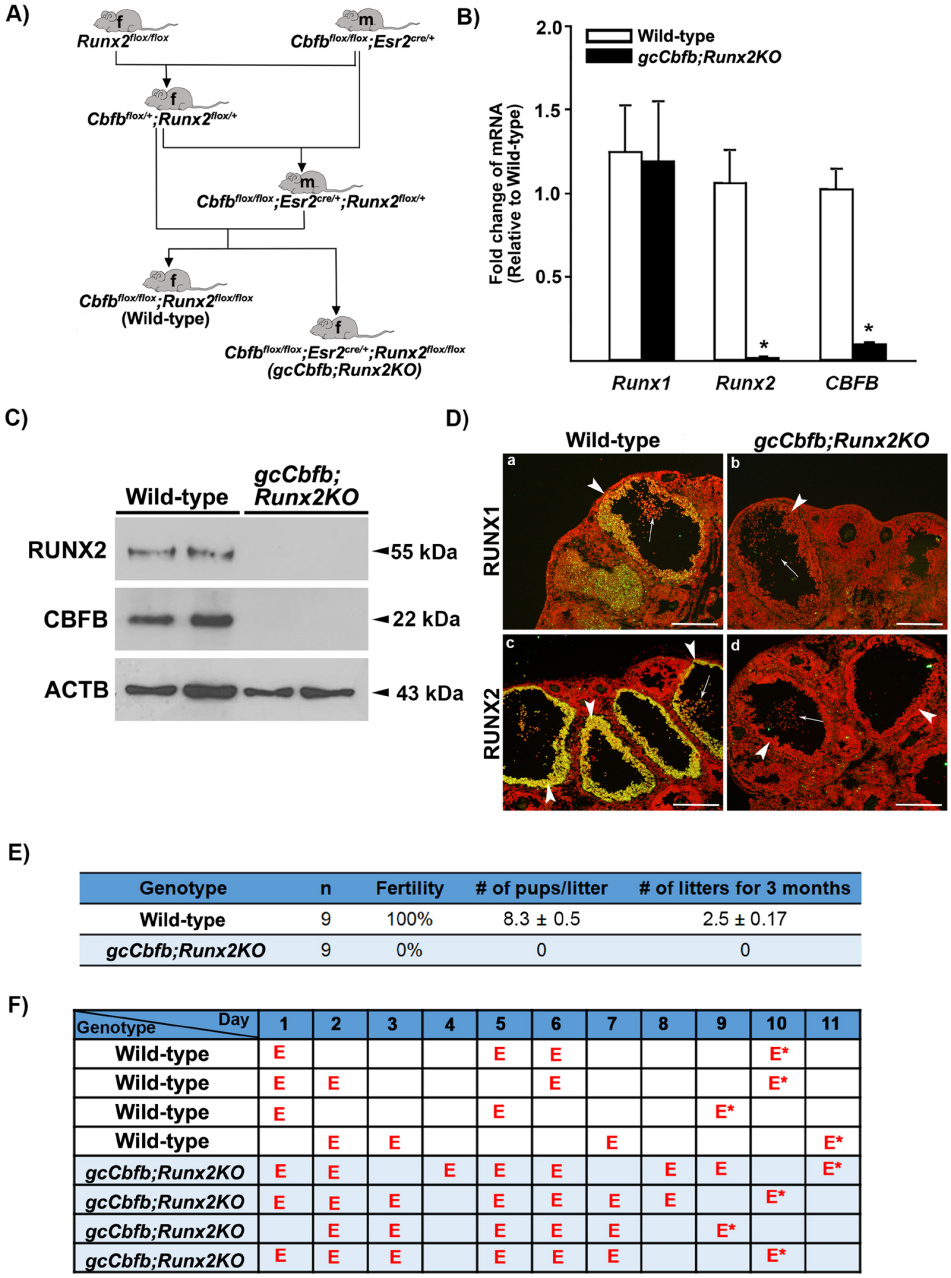
*Cbfb*^*flox/flox*^; *Esr2*^*cre/+*^*;Runx2* ^*flox/flox*^ (*gcCbfb;Runx2KO*) mice: Generation and verification of ovarian ablation of *Runx2* and *Cbfb* expression and assessment of fertility and estrous cycling pattern. (**A**) Breeding schemes used to generate *Cbfb*^*flox/flox*^; *Esr2*^*cre/+*^*;Runx2* ^*flox/flox*^ (*gcCbfb;Runx2KO*) and *Cbfb*^*flox/flox*^;*Runx2* ^*flox/flox*^ (Wild-type) mice are depicted. F and M in the shape of mice represent female and male, respectively. (**B**) Granulosa cells were isolated from ovaries collected at 11 h post-hCG from PMSG-primed immature mice. Levels of mRNA for *Runx1, Runx2*, and *Cbfb* were measured by qPCR and normalized to the *Rpl19* value in each sample (n = 8 animals/genotype). *p < 0.0001. (**C**) Western blot detection of RUNX2 and CBFB proteins in granulosa cells isolated at 11 h post-hCG. The membrane was re-probed with a monoclonal antibody against beta-actin (ACTB) to assess the loading of protein in each lane (n = 2 animals/genotype). (**D**) Immunohistochemical detection of RUNX1 and RUNX2 in ovaries of control and mutant mice collected at 11 h post-hCG (n = 3 animals/genotype). Immune-positive staining for RUNX1 or RUNX2 proteins (yellow/green) was localized to granulosa cells of periovulatory follicles in wild-type mice. Arrowheads and arrows point to granulosa cells and cumulus cells of preovulatory follicles, respectively. The sections were counterstained with propidium iodide (red) for nuclear staining. Scale bars, 250 μm for all images. (**E**) At 2 months of age, wild-type and *gcCbfb;Runx2KO* mice were mated with fertile males for 3 months. (**F**) Estrus cyclicity was monitored by daily vaginal smears for at least 2 cycles, and the presence of predominantly cornified cells was labeled as E (estrus). The animals were euthanized in the morning of estrus and marked E*.

Female *Cbfb*^*flox/flox*^*;Esr2*^*Cre/+*^ mice were subfertile^10^. Therefore, to generate a mutant mouse line bearing granulosa cell deletion of both the *Runx2* and *Cbfb* gene (*Cbfb*^*flox/flox*^*;Esr2*^*Cre/+*^*;Runx2*^*flox/flox*^, referred as *gcCbfb;Runx2KO*), the breeding scheme depicted in Fig. 1A was used by first mating female *Runx2*^*flox/flox*^ with male *Cbfb*^*flox/flox*^*;Esr2*^*Cre/+*^ mice. To verify the ablation of *Cbfb* and *Runx2* expression, granulosa cells were isolated from ovaries of immature mice at 11 h after hCG administration. The levels of mRNA for *Runx2* and *Cbfb*, but not for *Runx1*, were drastically reduced in *gcCbfb;Runx2KO* mice compared to *Runx2* ^*flox/flox*^*;Cbfb* ^*flox/flox*^ (wild-type) mice (Fig. 1B). Similarly, RUNX2 and CBFB were undetectable in granulosa cells of *gcCbfb;Runx2KO* mice (Fig. 1C). Immunohistochemical analyses further confirmed the ablation of RUNX2 in preovulatory follicles of the double KO mice (Fig. 1D-d). In addition, little staining for RUNX1 was detected in the preovulatory follicles of this mutant mouse (Fig. 1D-b). This is consistent with a previous report showing that in the absence of *Cbfb*, RUNX1 protein became unstable and was likely ubiquitinated in granulosa cells of periovulatory follicles^10^. These data confirmed the deletion of *Runx2* and *Cbfb* as well as the loss of RUNX1 protein in granulosa cells of preovulatory follicles in mutant mice.

### *gcCbfb;Runx2KO* mice are infertile and display disturbed estrous cycles

To determine the reproductive capacity, female wild-type and *gcCbfb;Runx2KO* mice at two months of age were subjected to a fertility test by mating with males of proven fertility at least for three months (Fig. 1E). None of the double KO mice produced pups, while wildtype animals were all fertile. After the end of the breeding trial, these animals were subjected to daily vaginal cytology for at least two cycles. The double KO mice stayed longer in estrus (marked as E, the presence of predominantly cornified epithelial cells) than wild-type mice (72% vs. 37%), indicative of irregular estrous cycles (Fig. 1F). Of note, the breeding scheme used (Fig. 1A) also produced mutant mice deficient of the *Runx2* gene in granulosa cells (*Cbfb*^*flox/+*^*;Esr2*^*Cre/+*^*;Runx2*^*flox/flox*^, referred to *gcRunx2KO*). These mice bear a heterozygous deletion of *Cbfb* in granulosa cells. *gcRunx2KO* mice showed complete ablation of RUNX2 but displayed the normal expression of RUNX1 in granulosa cells of periovulatory follicles (Supplementary Fig. 1A). These mutant mice displayed the subfertility phenotype; three out of six female mice that were mated with fertile males became pregnant and/or delivered pups (3, 4, 6 pups for each), while the remaining three did not show any sign of pregnancy during the 2 months trial period (Supplementary Fig. 1B).

### *gcCbfb;Runx2KO* mice failed to ovulate

**Figure 2:**
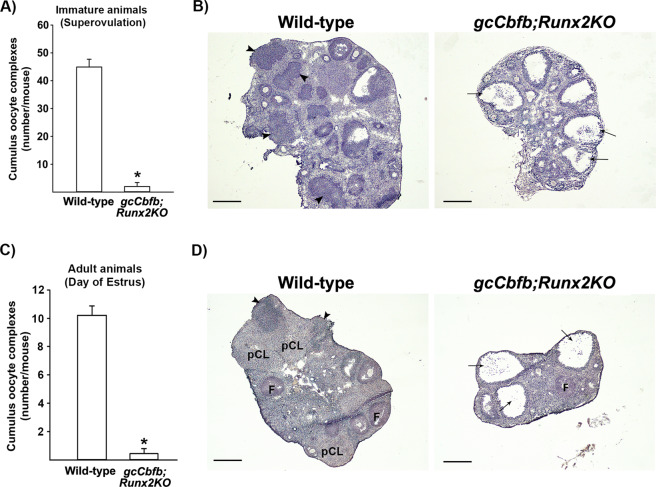
*gcCbfb;Runx2KO* mice failed to ovulate. (**A & B**) Immature wild-type and *gcCbfb;Runx2KO* mice primed with PMSG/hCG were killed between 16–24 h after hCG administration and cumulus oocyte complexes (COCs) were collected from oviducts and counted (A), and their ovarian morphology was examined (**B**) (n = 13 and 8 for control and mutant mice, respectively). *p < 0.001. (**C & D**) Adult mice were euthanized in the morning of estrus (marked as E* in Fig. 1F). COCs were collected from oviducts and counted (**C**), and their ovarian morphology was examined (**D**) (n = 5 and 9 for control and mutant mice, respectively). *p < 0.001. Arrows and arrowheads point to large antral follicles with expanded cumulus cells and newly forming CL, respectively. pCL; CL generated from previous cycles. F; follicle. Scale bars, 500 μm for all images.

To determine whether the ovulatory capacity was compromised in mutant mice, ovulation rates and ovarian morphology were assessed using both superovulation-induced immature mice and unstimulated adult animals. The ovulation-induced model allowed us to bypass potential impacts of *Esr2 Cre* expression in non-ovarian tissues (e.g., hypothalamus/pituitary). Examination of the oviduct of superovulation-induced mice showed significantly lower numbers of oocytes released in double knockout mice (6 out of 8 mice had no COCs) compared to that in wild-type animals (Fig. 2A). Histology of the ovary obtained 16 h post-hCG showed the presence of expanded cumulus cells and oocytes entrapped in large follicles in the double KO mice (Fig. 2B), whereas the ovary of wild-type animals displayed many newly forming CLs. The ovulation rate was also reduced (~70%) in *gcRunx2KO* mice compared to wild-type animals (Supplementary Fig. 1C). Ovaries of *gcRunx2KO* mice displayed multiple large follicles with entrapped COCs as well as forming CLs after superovulation-induction (Supplementary Fig. 1D).

For adult animals, their ovaries and oviducts were collected in the morning (between 1100 to 1200 h) of estrus (marked as E* in Fig. 1F). This is the time point when oocytes can be collected in the oviduct because ovulation typically occurs in the early morning of estrus, and newly formed CLs can be easily identified histologically in the ovary. As expected, an average of 10 oocytes was collected in oviducts of wild-type animals, whereas *gcCbfb;Runx2KO* mice had only a few oocytes if any in the oviduct (7 out of 9 mice had no COCs)(Fig. 2C). As expected, in wild-type animals, multiple newly forming CLs, as well as CLs generated from previous cycles, were found in the ovary (Fig. 2D). Meanwhile, the double KO mice showed multiple large antral follicles, some of them with entrapped COCs, but no CL (Fig. 2D). These differences were strikingly similar to those observed with the superovulation model, indicating that the infertility phenotype of mature double KO mice was mainly caused by anovulation and failure to develop functional CL.

### Identification of genes whose expression was affected in the *gcCbfb;Runx2KO* mouse ovary

To determine the mechanisms causing impaired ovulation in mutant mice, ovaries of immature animals collected at 3 and 11 h post-hCG were used to analyze the expression of key ovulatory genes. These time points were selected to determine whether anovulatory phenotype was caused by defects in early ovulatory response (e.g., failure to respond to hCG/LH stimulation) or late ovulatory events via assessing early and late changes in gene expression, respectively. The levels of mRNA for *Areg*, *Ereg*, *Fos*, *Pgr*, and *Ptgs2* were compared between control and mutant mouse ovaries obtained at 3 h post-hCG. We selected these genes as hCG rapidly induces their expressions in preovulatory follicles within a few hours^4,29,30^. Moreover, these genes are known to be critical for ovulation^4,29,30^.

**Figure 3:**
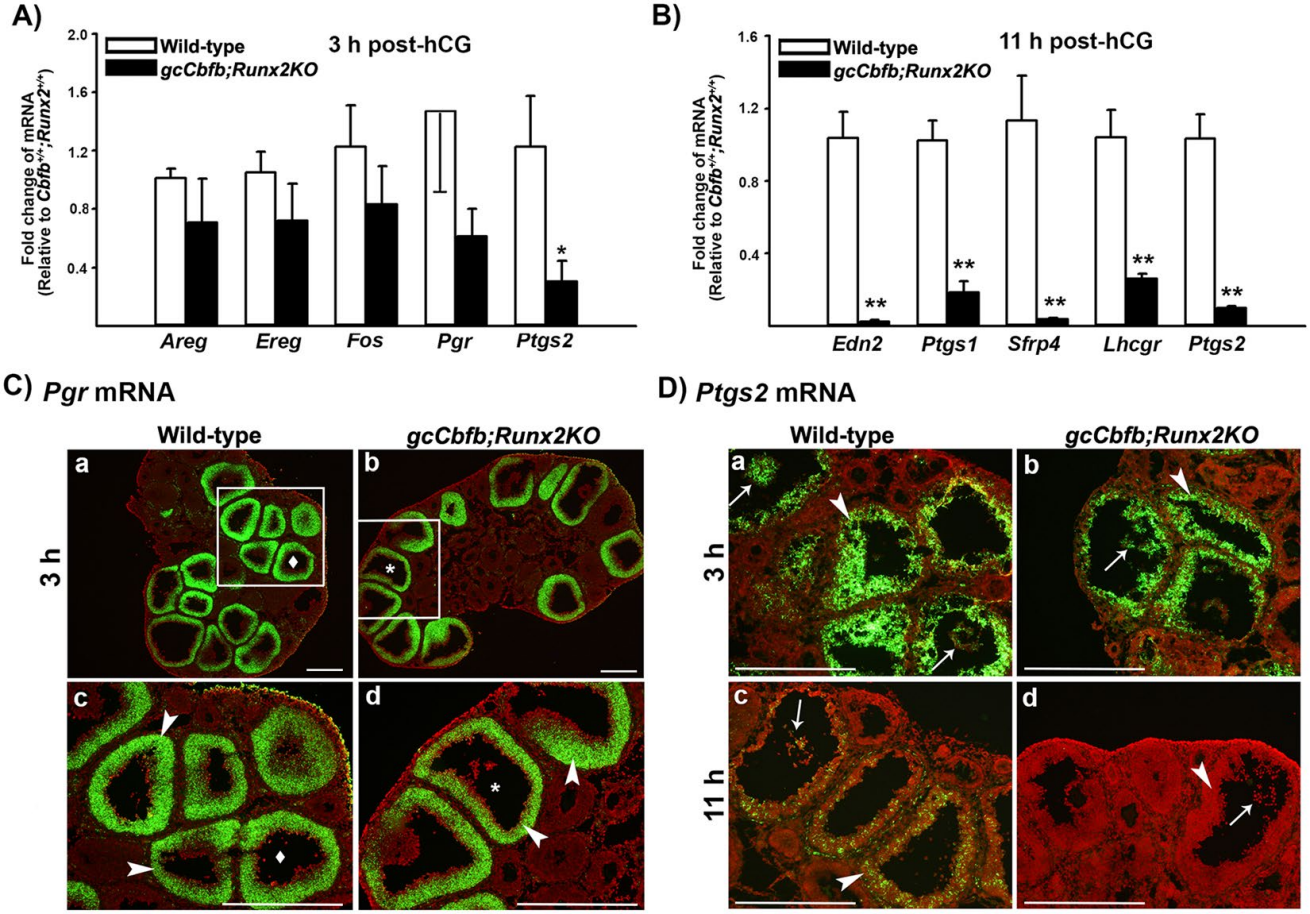
The differential expression of key early and late ovulatory genes in ovaries of *gcCbfb;Runx2KO* mice. (**A**) Ovaries were collected at 3 h after hCG administration. The levels of mRNA for early ovulatory genes were measured by qPCR, normalizing to the *Rpl19* value in each sample (n = 7 and 6 for control and mutant mice, respectively). *p < 0.05. (**B**) Ovaries were collected at 11 h after hCG administration. The levels of mRNA for late ovulatory genes were measured by qPCR, normalizing to the *Rpl19* value in each sample (n = 8 and 7 for control and mutant mice, respectively). **p < 0.01. (**C & D**) The localization of *Pgr* mRNA and *Ptgs2* mRNA was evaluated via *in situ* hybridization analyses in ovaries collected at 3 and 11 h post-hCG (n = 3/genotype). Green fluorescence staining represents transcripts for these genes. The tissue sections were counterstained with propidium iodide (red). Arrowheads and arrows point to granulosa cells of preovulatory follicles and expanded cumulus cells, respectively. Scale bars, 500 μm for all the images.

The levels of mRNA for *Areg*, *Ereg*, *Fo*s, and *Pgr* were not different between wild-type mice and *gcCbfb;Runx2KO* mice, while the level of *Ptgs2* mRNA was slightly lower in the ovary of *gcCbfb;Runx2KO* mice (Fig. 3A). Since *Ptgs2* expresses in both granulosa and cumulus cells, we determined whether the expression of *Ptgs2* affected in the specific compartment in the follicle by *in situ* hybridization analyses (ISH). The localization of *Pgr* mRNA was also assessed to verify that this gene is up-regulated in individual preovulatory follicles comparable to wild-type animals. Indeed, *Pgr* mRNA was localized to granulosa cells of many preovulatory follicles in both wild-type and *gcCbfb;Runx2KO* mice. Similarly, *Ptgs2* mRNA was localized to both granulosa and cumulus cells in both animal models, but there were no visible differences in the intensity and localization pattern in the ovary collected at 3 h after hCG administration (Fig. 3C). These data indicated that the double knockout mice were able to induce the expression of early key ovulatory genes in response to hCG stimulation similar to those observed in wild-type mice. This notion was further supported by our pilot study showing that *Lhcgr* mRNA was localized to granulosa and theca cells in the ovary of double KO mice collected before hCG administration (Supplementary Fig. 3A). Similarly, *Cyp19a1* mRNA was also localized to granulosa cells (Supplementary Fig. 3B). Together, these data indicated that *Lhcgr* and *Cyp19a1* were expressed in granulosa cells of follicles in the ovary of double KO mice during follicular development in a manner similar to those in wild-type mice.

In the previous study using *gcCbfbKO* mice, we reported that the levels of mRNA for *Edn2*, *Ptgs1*, *Lhcgr*, and *Sfrp4* were lower in granulosa cells collected at 12 h post-hCG compared to those in wild-type animals^10^. In the current study, we also confirmed that the levels of mRNA for all these genes were significantly down-regulated in the ovary of double knockout mice at 11 h post-hCG (Fig. 3B). Because *Ptgs2* mRNA levels were reported to have a second peak at 12 h after hCG administration in mice^31^, we compared mRNA for this gene and found that the levels were significantly lower (Fig. 3B). Consistently, *ptgs2* mRNA was barely visible in double KO mouse ovaries at 11 h post-hCG, as demonstrated by *In situ* hybridization analysis (Fig. 3D). Taken together, these data suggested that double KO mice displayed marginal morphological and gene expression changes during the early ovulatory period while exhibiting more profound changes in gene expression during the late ovulatory period.

Thus far, we have identified three genes, *Edn2*, *Ptgs1*, and *Ptgs2*, that were directly linked to the ovulatory process and significantly down-regulated in *gcCbfb;Runx2KO* mice. To further identify ovulatory genes whose expression was affected in this anovulatory mutant mice, total RNA isolated from the ovaries collected at 11 h post-hCG was used for RNA-seq analyses (n = 4 animals/ genotype). Differential gene expression analysis on RNA-seq data revealed 715 and 293 genes as being down- and up-regulated in mutant mice compared to control mice, respectively (*q* < 0.05). The genes meeting the further criteria of the fold change of 2 or higher and average expression value of 0.5 or higher are listed in Supplementary Table 2 (342 genes met these criteria). These criteria were chosen to identify genes with both high expression levels and dramatic changes. Some of the most highly down- or up-regulated genes and the genes that are known to be involved in ovulation are listed in Tables 1 and 2. Included in these RNA-seq data and verified by qPCR are the genes that were previously found to be differentially regulated in cultured granulosa cells of *Cbfb*^*flox/flox*^ x *Cyp19*^*cre*^ mice, including *Edn2*, *Ptgs1, Sfrp4*, and *Lhcgr* (marked with * in Tables 1 and 2 and Supplementary Table 2).

**Table 1:** A list of selected down-regulated genes in *gcRunx2;CbfbKO* mice.

Official Gene Name	Fold change
***Edn2*** (endothelin 2)*	−49.22
***Cldn18*** (claudin 18)	−16.89
***Sfrp4*** (secreted frizzled-related protein 4)*	−15.12
***Wnt10b*** (wingless-type MMTV integration site family, member 10B)	−14.37
***Has1*** (hyaluronan synthase 1)	−12.84
***Il11*** (interleukin 11)	−11.89
***Il6*** (interleukin 6)	−11.86
***Serpine1*** (serine (or cysteine) peptidase inhibitor, clade E, member 1)	−9.82
***Akr1c18*** (aldo-keto reductase family 1, member C18)	−9.37
***Xpnpep2*** (X-prolyl aminopeptidase (aminopeptidase P) 2, membrane-bound)	−8.67
***Spp1*** (secreted phosphoprotein 1)	−8.23
***Rtl1*** (retrotransposon Gaglike 1)	−8.04
***Kl*** (klotho)	−7.70
***Rnf125*** (ring finger protein 125)	−7.59
***Cemip*** (cell migration inducing protein, hyaluronan binding)*	−7.22
***Mmel1*** (membrane metallo-endopeptidase-like 1)*	−7.12
***Lrp8*** (low density lipoprotein receptor-related protein 8, apolipoprotein e receptor)*	−6.61
***Fosb*** (FBJ osteosarcoma oncogene B)	−6.31
***Prkg2*** (protein kinase, cGMP-dependent, type II (PGR downstream)	−6.29
***Ankrd1*** (ankyrin repeat domain 1 (cardiac muscle))	−6.27
***Ptgfr*** (prostaglandin F receptor)*	−6.09
***Btg2*** (B cell translocation gene 2, anti-proliferative)	−5.72
***Ptgs2*** (prostaglandin-endoperoxide synthase 2)	−5.66
***Fosl1*** (fos-like antigen 1)	−5.50
***F3*** (coagulation factor III)	−5.40
***Fabp4*** (fatty acid binding protein 4, adipocyte)	−4.92
***Apln*** (apelin)*	−4.86
***Hp*** (haptoglobin)*	−4.83
***Ptgs1*** (prostaglandin-endoperoxide synthase 1)*	−4.74
***Adamts1*** (a disintegrin-like and metallopeptidase with thrombospondin type 1 motif, 1)	−4.34
***Lhcgr*** (luteinizing hormone/choriogonadotropin receptor)*	−3.64
***Sgk1*** (serum/glucocorticoid regulated kinase 1)*	−3.31
***Parm1*** (prostate androgen-regulated mucin-like protein 1)	−3.24
***Junb*** (jun B proto-oncogene)	−2.99
***Ctsl*** (cathepsin L)	−2.88
***Adamts4*** (a disintegrin-like and metallopeptidase with thrombospondin type 1 motif, 4)	−2.78
***Fos*** (FBJ osteosarcoma oncogene)	−2.47
***Ccrl2*** (chemokine (C-C motif) receptor-like 2)*	−2.47
***Timp1*** (tissue inhibitor of metalloproteinase 1)*	−2.39
***Adamts5*** (a disintegrin-like and metallopeptidase with thrombospondin type 1 motif, 5)	−2.10

**Table 2:** A list of selected up-regulated genes in *gcRunx2;CbfbKO* mice.

Official Gene Name	Fold change
*Hsd17b1* (hydroxysteroid (17-beta) dehydrogenase 1)*	2.72
*Vcam1* (vascular cell adhesion molecule 1)	2.91
*Inhbb* (inhibin beta-B)*	4.30
*Grem1* (gremlin 1, DAN family BMP antagonist)*	4.46
*Cyp17a1* (cytochrome P450, family 17, subfamily a, polypeptide 1)	6.16
*Gpr83* (G protein-coupled receptor 83)	9.71

^*^, genes which were previously identified to be differentially regulated in cultured granulosa cells isolated from *Cbfb*^*flox/flox*^*;Cyp19*^*cre*^ mice compared to those from wild-type mice^9^.

Fold changes were calculated by comparing the average values for mutant mouse samples vs. littermate control mouse samples (*gcRunx2;CbfbKO*/Wild-type). Lower expression in mutant mouse samples is given as −2 rather than 0.5. The genes verified by qPCR were highlighted in green and red in Table 1 and Table 2, respectively.

**Figure 4:**
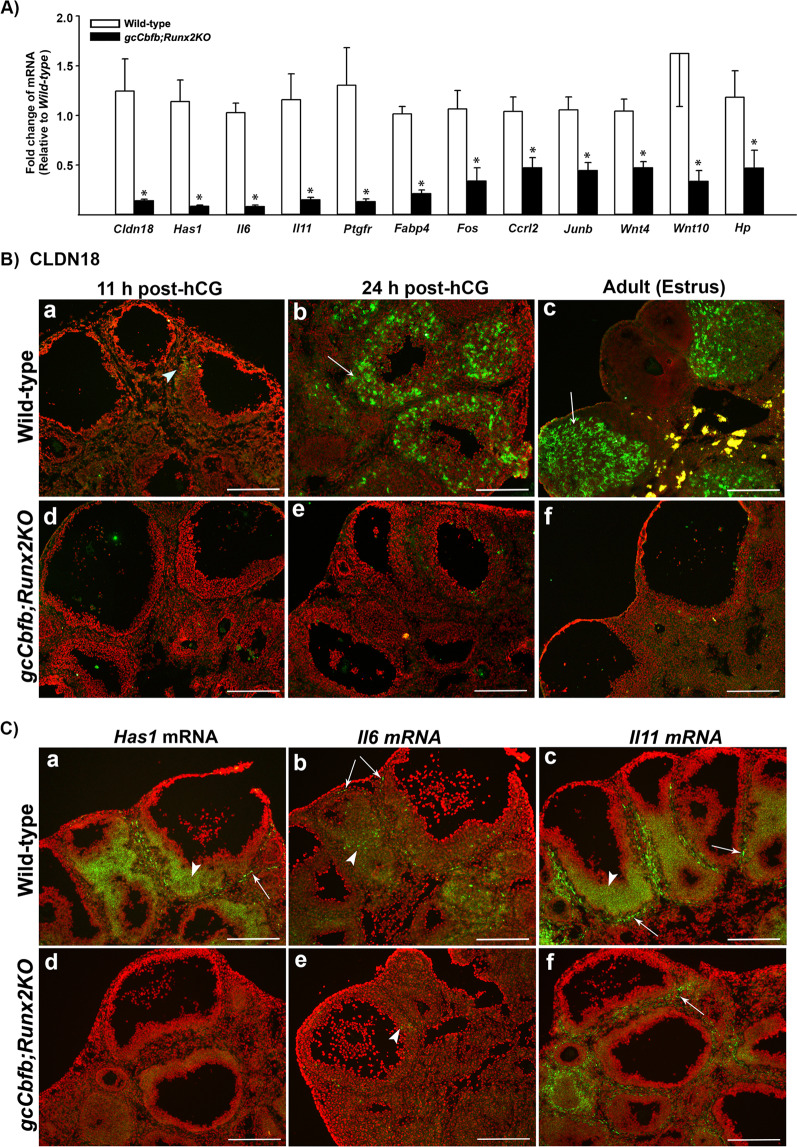
Identification of down-regulated genes in ovaries of *gcCbfb;Runx2KO* mice (I). (**A**) Ovaries were collected at 11 h after hCG administration from wild-type and *gcCbfb;Runx2KO* mice. A list of the most highly downregulated genes was selected from RNA-seq data analyses. The levels of mRNA for genes were measured by qPCR, normalizing to the *Rpl19* value in each sample (n = 8 and 7 for control and mutant mice, respectively). *p < 0.01. (**B**) Ovaries were collected at 11 and 24 h after hCG administration from immature animals as well as in the morning of estrus from adult mice. The localization of CLDN18 was evaluated via immunohistochemical analyses (n = 3/genotype). CLDN18 was detected as green fluorescent staining, and the tissue sections were counterstained with propidium iodide (red). Arrowheads and arrows point to a few granulosa cells and luteal cells stained positively for CLDN18, respectively. (**C**) *in situ* hybridization analyses were used to localize mRNA for *Has1*, *IL6*, and *IL11* in ovaries collected at 11 h post-hCG (n = 3/genotype). Transcripts for these genes were detected as green fluorescence signals. The tissue sections were counterstained with propidium iodide (red). Arrowheads and arrows point to granulosa cells of preovulatory follicles and cells in the theca layer positive for these gene transcripts, respectively. Scale bars, 250 μm for all the images.

Because many genes identified in RNA-seq analysis have not previously reported being expressed in the ovary and/or regulated by CBFs, we have also selected a group of genes that were highly affected in the ovary of *gcCbfb;Runx2KO* mice for qPCR verification (Fig. 4A). Notable among the most highly down-regulated genes were *Cldn18*, *Has1*, *Il11*, and *Il6* (Fig. 4A). CLDN18 is a tight junction molecule^32,33^. However, the ovarian expression of this protein is not known. The immunopositive staining for CLDN18 was localized to a few small patches of the granulosa cell compartment in periovulatory follicles at 11 h post-hCG, yet it was abundant in newly forming CLs in wild-type mouse ovaries (Fig. 4B-a & b). No staining was observed at 0 h post-hCG (data not shown). In wild-type adult mice, CLDN18 was localized to all CLs (Fig. 4B-c). In contrast, little staining for CLDN18 was detected in preovulatory and unruptured follicles of the double KO mice (Fig. 4B-d,e,f). HAS1 is an enzyme that synthesizes hyaluronic acid^34^. *In situ* hybridization analyses showed that *Has1* mRNA was localized to cells in the theca layer and granulosa cells of preovulatory follicles in wild-type mouse ovaries, and this expression was diminished in the ovary of the mutant mice (Fig. 4C-a,d). Similarly, *Il6* mRNA and *Il11* mRNA were localized to the granulosa cell layer of preovulatory follicles and the theca layer, albeit with different intensity (Fig. 4C-b,c). As expected, the expression of these genes was down-regulated in the double KO mouse ovary.

**Figure 5:**
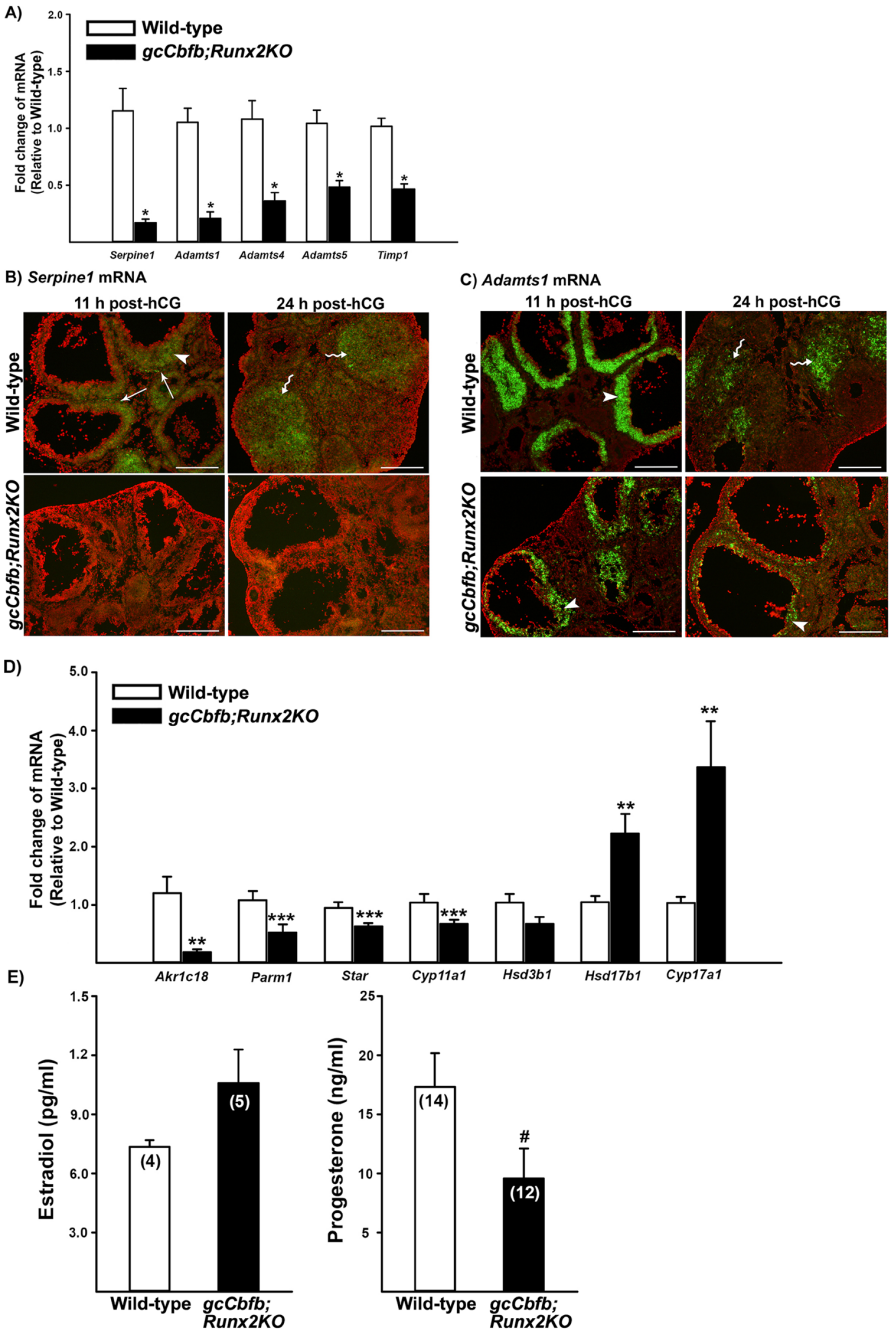
Identification of down-regulated genes in ovaries of *gcCbfb;Runx2KO* mice (II). Ovaries were collected at 11 h after hCG administration from wild-type and *gcCbfb;Runx2KO* mice. (**A**) The levels of mRNA for a cluster of genes associated with tissue remodeling were measured by qPCR, normalizing to the *Rpl19* value in each sample (n = 8 and 7 for control and mutant mice, respectively). *p < 0.005. (**B & C**) The localization of mRNA for *Serpine1* and *Adamts1* was detected via *in situ* hybridization analyses in ovaries collected at 11 and 24 h post-hCG (n = 3/genotype). Transcripts for these genes were detected as green fluorescence signals. The tissue sections were counterstained with propidium iodide (red). Arrowheads, arrows, and wavy arrows point to granulosa cells of preovulatory follicles, cells in the theca layer, and luteal cells in the corpus luteum stained positive for these gene transcripts, respectively. Scale bars, 250 μm for all the images. (**D**) The levels of mRNA for genes known to be involved in steroid metabolism were measured by qPCR, normalizing to the *Rpl19* value in each sample (n = 8 and 7 for control and mutant mice, respectively. **p < 0.01. ***p < 0.05. **E**) The levels of serum estradiol and progesterone were measured in blood samples collected at 11 h after hCG administration. The number inside each bar represents the sample size of mice evaluated. ^**#**^*p* = 0.056.

In addition, the list of differentially regulated genes included clusters of genes involved in tissue remodeling (e.g., *Serpine1, Mmel1, Adamts1, Cts1, Adamts4, Adamts5*, and *Timp1*) and steroid metabolism (e.g., *Ark1c18*, *Parm1*, *Hsd17b11*, *Hsd3b6*, *Hsd17b1*, and *Cyp17a1*). Using qPCR analyses, we verified the downregulated expression of *Serpine1, Adamts1, Adamts4, Adamts5*, and *Timp1* between and control mouse ovaries (Fig. 5A). *In situ* hybridization further demonstrated the localization of *Serpine1* and *Adamts1* mRNA in preovulatory follicles and newly forming CL in control animals and the down-regulated expression of these genes in the ovary of *gcCbfb;Runx2KO* mice (Fig. 5B,C). qPCR analyses of genes involved in progesterone synthesis and metabolism showed that the levels of mRNA for *Ark1c18*, *Parm1*, *Star*, and *Cyp11* were lower, whereas the levels of mRNA for *Hsd17b1* and *Cyp17a1* were higher in *gcCbfb;Runx2KO* mouse ovaries than those in wild-type mouse ovaries (Fig. 5D). Similarly, serum progesterone levels were modestly decreased in double KO mice, but not statistically significant (*p* = 0.056), whereas estradiol levels were not different between wild-type and mutant mice at 11 h post-hCG (Fig. 5E).

### *gcCbfb;Runx2KO* mice failed to develop normal CL

**Figure 6:**
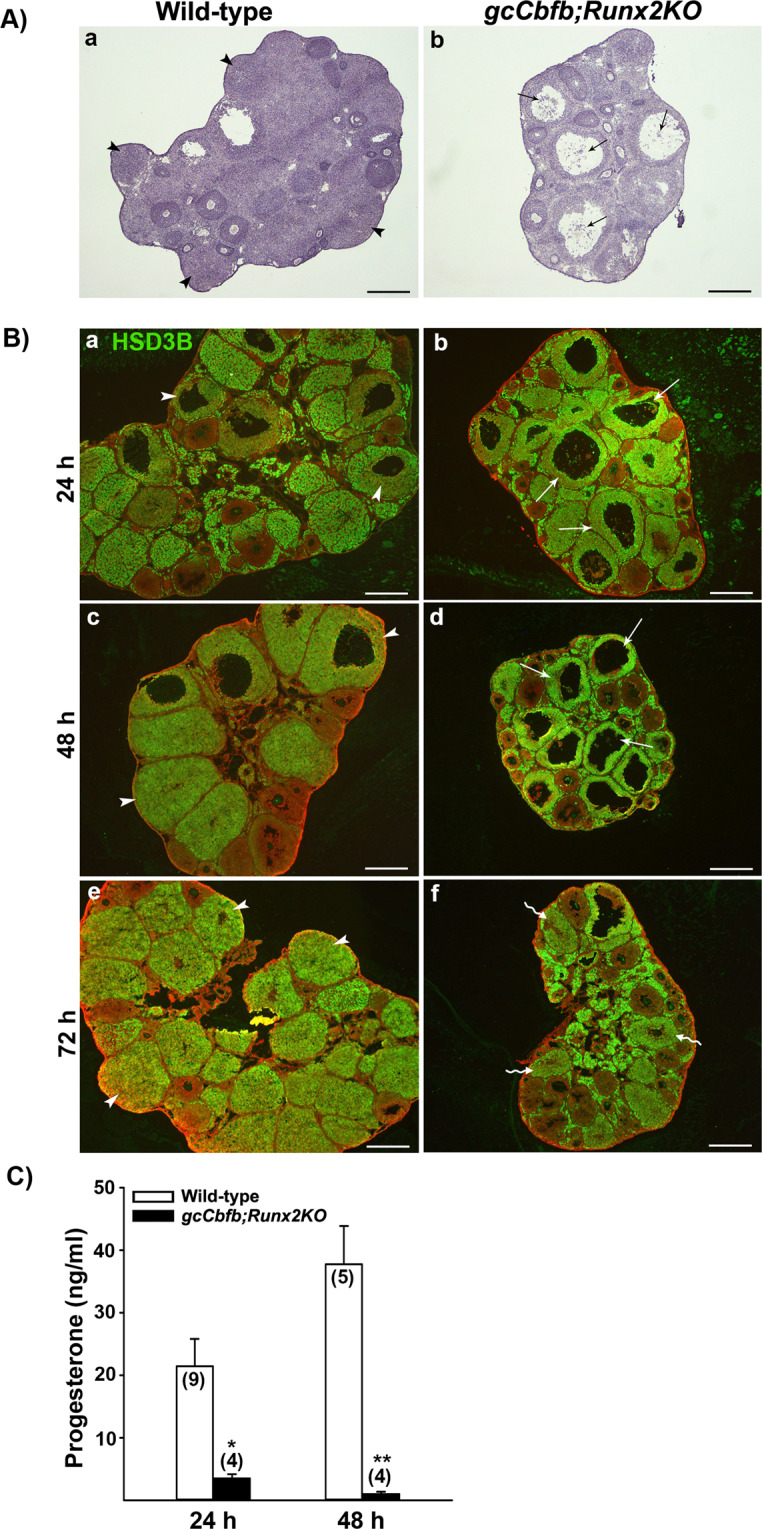
Assessment of ovarian morphology during the post-ovulatory period in *gcCbfb;Runx2KO* mice. (**A**) Ovarian sections obtained at 24 h post-hCG were stained with hematoxylin (n = 3 animals/ genotype). (**B**) Ovarian sections obtained at 24, 48, and 72 h post-hCG were immunostained for HSD3B to easily locate corpora lutea and unruptured follicles (n = 3 animals/genotype). HSD3B was detected as green fluorescent staining, and ovarian sections were counterstained with propidium iodide (red). Arrowheads, arrows, and wavy arrows point to corpora lutea, unruptured follicles, and collapsed luteinized follicles, respectively. Scale bars, 500 μm for all the images. (**C**) The levels of serum progesterone were measured in blood samples collected at 24 and 48 h after hCG administration. The number inside each bar represents the number of mice evaluated. *p = 0.024, **p = 0.001.

*gcCbfb;Runx2KO* mice developed large preovulatory follicles but failed to ovulate in response to hCG or the LH surge. To determine whether these follicles develop into the CL, we examined ovaries collected at 24, 48, and 72 h after hCG administration. At 24 h, ovaries of wild-type animals showed many forming CLs (Fig. 6A-a), while the ovary of double KO mice exhibited multiple large antral follicles, each with expanded COCs (Fig. 6A-b). To mark the CL and preovulatory follicles, ovarian sections were immunostained with HSD3B, an enzyme that catalyzes pregnenolone to progesterone (Fig. 6B). In wild-type animals, ovarian sections from post-ovulation time points showed typical CL development. In contrast, ovarian sections of *gcCbfb;Runx2KO* mice showed multiple unruptured follicles even at 48 h post-hCG. By Day 72 h, no longer were these follicles present, but remained only small luteinized structures (Fig. 6B-f, wavy arrows). Consistent with these data, serum progesterone levels were completely diminished by 48 h post-hCG (Fig. 6C).

**Figure 7:**
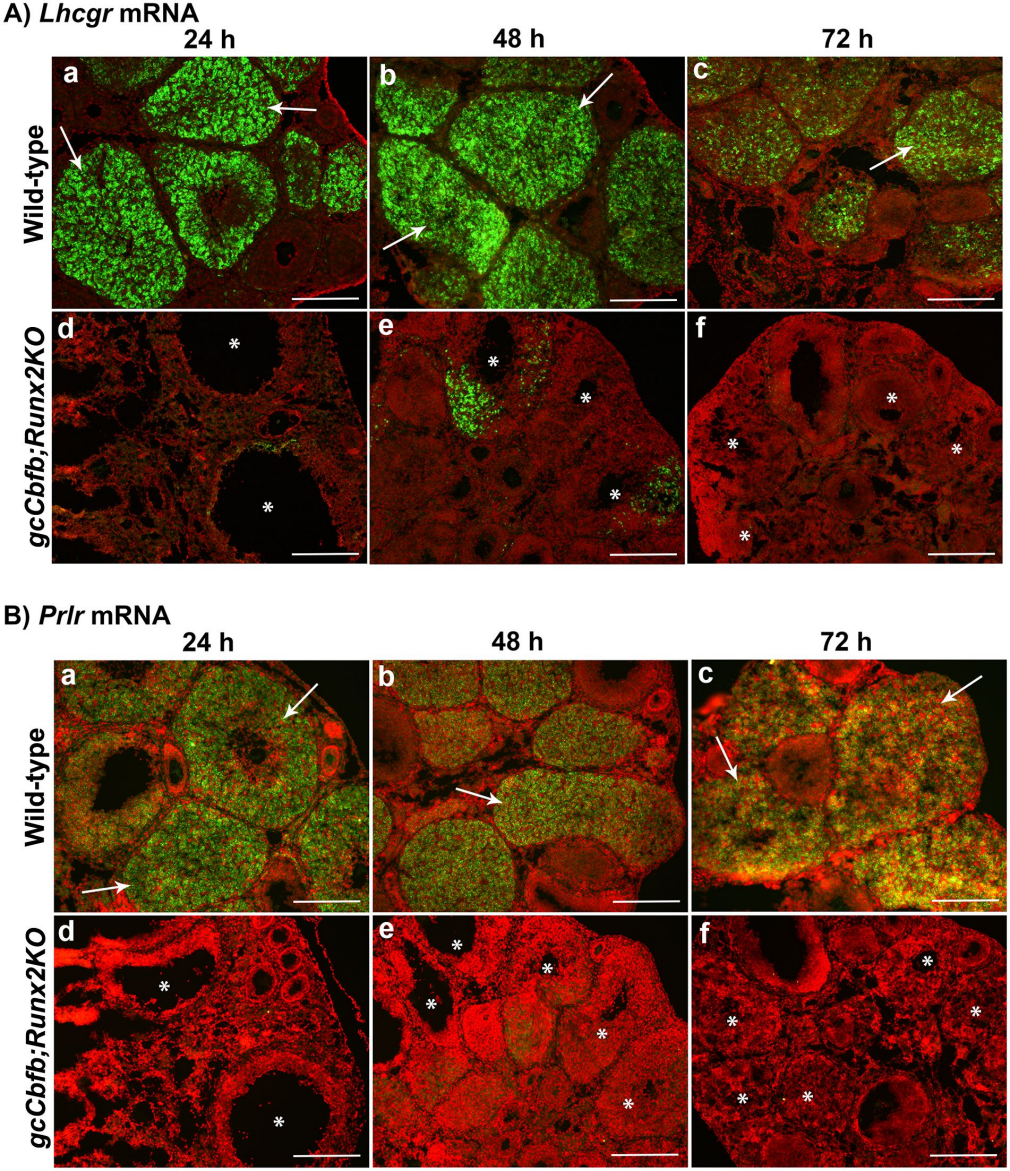
Assessment of the expression of *Lhcgr* and *Prlr* mRNA during the post-ovulatory period in *gcCbfb;Runx2KO* mice. Ovaries collected at 24, 48, and 72 h after hCG administration were used to examine the expression of mRNA for *Lhcgr* (**A**) and *Prlr* (**B**) by *in situ* hybridization analyses (n = 3 animals/genotype). Transcripts for each gene were detected as green fluorescence signals. The ovarian sections were counterstained with propidium iodide (red). Arrows point to the CL expressing *Lhcgr* and *Prlr* mRNA in control animals, and the symbol (*) was placed in unruptured follicles and poorly luteinized collapsed follicles in *gcCbfb;Runx2KO* mice. Scale bars, 250 μm for all the images.

**Figure 8:**
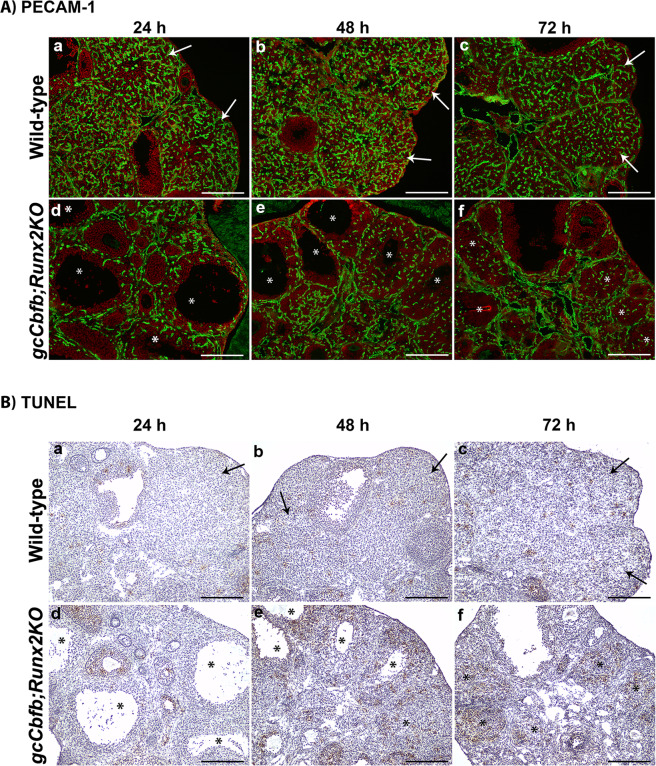
Assessment of vascularization and apoptosis during the post-ovulatory period in *gcCbfb;Runx2KO* mice. Ovaries were collected at 24, 48, and 72 h after hCG administration (n = 3 animals/ genotype). (**A**) Immunohistochemical analysis was used to detect PECAM-1 (green fluorescence staining). The ovarian sections were counterstained with propidium iodide (red). (**B**) TUNEL assay was used to detect apoptotic cells (brown staining). The ovarian sections were counterstained with hematoxylin (blue). Arrows point to CLs in control animals and the symbol (*) was placed in unruptured follicles and poorly luteinized collapsed follicles in *gcCbfb;Runx2KO* mice. Scale bars, 250 μm for all the images.

To investigate why periovulatory follicles failed to develop into the CL, we examined the expression of mRNA for *Lhgcr* and *Prlr*, two genes that are known to be essential for luteal development. The abundant expression for both *Lhcgr* and *Prlr* mRNA was localized to CLs at all three post-ovulatory time points in wild-type animals (Fig. 7A,B, upper panels), but little to no expression was detected in unruptured follicles and unruptured-then-collapsed poorly luteinized structures in the double KO mice (Fig. 7A,B, lower panels). These unruptured follicles also showed limited staining for PECAM-1, an endothelial cell marker (Fig. 8A, lower panel), indicating the defective angiogenesis/vascularization during CL formation and development. These unruptured-then-collapsed follicles in *gcCbfb;Runx2KO* mouse ovaries were riddled with apoptotic cells as demonstrated by extensive TUNEL staining at 48 and 72 h post-hCG (Fig. 8B-e,f).

## Discussion

The present study is the first report showing that *gcCbfb;Runx2KO* mice displayed profound defects in both ovulation and CL development. The majority of the double knockout mice failed to ovulate (>95%) and develop functional CL, thus resulting in infertility. This anovulatory phenotype is likely the result of defects in the expression of specific genes, mainly in late ovulatory follicles. This notion is based on the findings that differences in the expression of key ovulatory genes were observed at 11 h post-hCG (e.g., *Edn2*, *Ptgs1*, *Ptgs2*), but not in the early ovulatory period (eg., *Areg*, *Ereg*, *Pgr*). In support of this, the morphology of preovulatory follicles appears to be indistinguishable between control and mutant mice at both 3 and 11 h after hCG administration. Only after the expected time of ovulation was the histological difference apparent. In wild-type animals, ovaries contained many newly forming CLs, whereas ovaries of the double KO mice showed multiple large antral follicles with entrapped expanding COCs, but no CLs.

The RNA-seq data analysis unveiled many genes whose expression was affected in the ovary of double KO mice during the periovulatory period. Notably, among the highly down-regulated genes are *Cldn18, Has1*, and *Il11*, whose expression has not been documented in the mouse ovary. CLDN18 is a tight junction protein that regulates paracellular permeability and architectural integrity in various epithelial cells^32,33^. The present study documented that hCG increased the expression of *Cldn18* in the mouse ovary, and the staining intensity of CLDN18 was highest in the forming CL. These data suggest that the primary role of CLDN18 is in CL formation, rather than the ovulatory process. HAS1 is an enzyme that synthesizes hyaluronan, a constituent of the extracellular matrix^34^. In the ovary, the expression of *Has2*, another member of hyaluronic acid synthases, increases in cumulus cells of preovulatory follicles^35^, and this up-regulation is critical for cumulus expansion^36^. Herein, we documented that *Has1* mRNA expression also increased in preovulatory follicles after hCG administration in the mouse ovary. Together, these data indicated that both *Has1* and *Has2* expression increases in preovulatory follicles, and these proteins play an important role in the ovulatory process by providing an essential component of the extracellular matrix. The next highly down-regulated genes in our double knockout mice were *Il11* and *Il6*, both of which are members of the IL6 type family of cytokines that share the common signal transducer receptor gp130^37^ and exert overlapping functions^38^. Previously, Liu *et al*.^39^ showed that the levels of *Il6* mRNA were increased in both granulosa cells and COCs after hCG stimulation, and the treatment of IL6/its soluble receptor (IL-6SR) induced the expansion of mouse COCs, proposing that IL6 could act as a potent autocrine mediator involved in COC expansion. Jang *et al*.^40^ documented the localization of *Il11* mRNA predominantly in the thecal layer of the rat ovary after hCG administration. In addition, the treatment of IL11 increased progesterone production in cultured rat preovulatory follicles^40^. Our current data demonstrated the localization of mRNA for *Il6* and *Il11* to granulosa and theca cells of preovulatory follicles and the compromised expression of these genes in the mutant mouse ovary. These data suggest that IL6 and IL11 are potential downstream targets of CBFs and are likely involved in the ovulatory process.

In addition to these genes, a cluster of genes involved in matrix remodeling was down-regulated in the double KO mouse ovary. Of these matrix remodeling genes, our particular interest was the down-regulated expression of *Adamts1*, *Cts1*, and *Adamts4*. These genes were reported to be down-regulated in *Pgr* knockout mice which failed to ovulate, but showed no visible defects in CL formation^4,41^, indicating that the reduced expression of these genes may contribute to the failure of follicle rupture in our mutant mice. Another group of genes differentially regulated were associated with steroid synthesis and metabolism. Interestingly, *Akr1c18* and *Parm1*, two highly down-regulated genes, have an opposite function in progesterone metabolism; AKR1C18 metabolizes progesterone to an inactive form^42–44^, whereas PARM1 inhibits progesterone metabolism to an inactive form^45^, suggesting that the impact on progesterone metabolism by these two genes could be canceled out in the double KO mice. Along with these two genes, there was a minor reduction in *Star* and *Cyp11a1* mRNA levels. In agreement with these findings, the progesterone levels were modestly decreased (*p* = 0.056, Fig. E) at 11 h after hCG administration. Meanwhile, the levels of mRNA for *Hsd17b1* and *Cyp17a1* were higher in the ovary of double mice. Since these genes are known to be involved in estradiol production^46,47^, we measured estradiol levels at both 3 and 11 h after hCG administration but found no difference between wild-type and mutant mice, suggesting that these changes were not sufficient to alter estradiol levels. It is also important to note that periovulatory progesterone levels were not altered in *gcCbfbKO* mice^10^, indicating that CBFs, likely through RUNX2, were involved in regulating the expression of selected genes associated with steroid metabolisms mentioned above. These changes also offered a glimpse into what would happen to periovulatory follicles after the expected time of ovulation in the double KO mouse ovary. In support of this notion, progesterone levels were further decreased at 24 h post-hCG and reached basal levels at 48 h post-hCG, consistent with the presence of poorly luteinized follicles at those time points.

The identification of these new genes differentially regulated in the double KO mice led to another question as to whether these genes mentioned above were uniquely regulated by RUNX2. We conducted preliminary studies comparing the expression of *Ptgs2*, *Cldn18*, and *Adamts1* in the periovulatory ovary collected from 4 different mouse models (e.g., wild-type, *gcCbfbKO, gcRunx2KO, gcCbfb;Runx2KO* mice). *In situ* hybridization and Immunohistochemical data showed that the expression of all three genes was also reduced in *gcRunx2KO* mice compared to that in wild-type and *gcCbfbKO* mice, although the most striking reduction was observed in the double KO mice (Supplementary Fig. 2). These data indicated that RUNX2 is likely a predominant regulator at least for these 3 genes in periovulatory follicles.

In addition to ovulatory defects, in *gcCbfb;Runx2KO* mice, periovulatory follicles never really transformed into the CL. Even 48 h post-hCG, the unruptured follicles maintained an antrum, and by 72 after hCG administration, these unruptured follicles collapsed and showed a sign of excessive apoptosis, suggesting that they eventually would be removed from the ovary by the apoptotic process. These defects were also strongly correlated with the lack of vascularization in the unruptured follicles even far beyond the ovulation timing. Vascular development/angiogenesis is controlled by a complex network of factors, encompassing growth factors and cytokines, cell adhesion molecules, and extracellular matrix proteinases and their inhibitors. Bioinformatic analysis (DAVID Bioinformatics Resources 6.8) on a list of down-regulated genes from our RNA-seq data generated several gene clusters linked to vascular development and angiogenesis. These include cytokines (*Il11*, *Il6*, *Clcf1*, *Cxcl1*, *Ngf, Tnfsf15, F3*), cell adhesion molecules (*Cldn18*, *H2-Q10*, *Nacam*, *Pdcd1*, *Sele*, *Spn*, *Sdc1*, *Sdc4*), and tissue remodeling proteinases and their inhibitors (*Serpine1*, *Timp1*, *Adamts1*, *Adamts4*, *Adamts5*, *Mmel1*, *Pappa*). Along with defects in vascular development, the failure to transform into the CL was correlated with the reduced expression of genes involved in luteinization. For instance, we verified the reduced levels for mRNA for *Lhcgr*, *Ptgfr*, *Ccrl2*, *Sfrp4*, *Wnt4*, and *Wnt10* in the ovary of mutant mice even before ovulation occurs. Throughout the next 3 days after ovulation, the unruptured large antral follicles failed to acquire the expression of *Lhcgr* and *Prlr*, receptors for the two most critical luteotropic hormones^48^, thus resulting in a rapid demise of poorly luteinized follicles in mutant mice.

It is also important to note that many of genes identified as differentially expressed in the ovary of *gcCbfb;Runx2KO* mice might not be direct transcriptional targets of RUNXs/CBFB, but rather their expressions were indirectly affected by the impact on the primary target of RUNXs/CBFB. Nevertheless, our RNA-seq data provide critical information that can be served as a foundation to identify the direct transcriptional target(s) of RUNXs/CBFB in periovulatory follicles.

In summary, we found that in *gcCbfb;Runx2KO* mice, ovulation and CL formation were completely inhibited, indicating that both RUNX1 and RUNX2 with their common partner CBFB in the ovary are required for successful ovulation and CL development. In support of this notion, *Runx2* deletion in granulosa cells (*gcRunx2KO* mice) resulted in a partial reduction in ovulation rates. Moreover, they appeared to have histologically normal-looking CL, indicating that unruptured follicles eventually transformed into the CL. In addition, the current RNA-seq data revealed that CBFs affect the expression of a diverse set of genes in ovulatory follicles and newly forming CL. Included in the list of genes are several clusters of genes associated with inflammation, matrix remodeling, cell differentiation, vascularization, and steroid metabolism. From *in vivo* data generated from the present study and our previous study^10^, we propose that the overlapping expression of *Runx1* and *Runx2* in ovulatory follicles not only brings about effective synergism required for successful ovulation and subsequent luteinization but also serves a necessary compensatory mechanism to ensure the success of these two critical events in the ovary.

## Materials and Methods

### Animals

All animals were treated in accordance with the National Institutes of Health Guide for the Care and Use of Laboratory Animals. Animal protocols were approved by the University of Kentucky Animal Care and Use Committees. *Cbfb*^*flox*^^17^, *Runx2*^*flox*^^49^, and *Esr2*^*Cre*^^50^ mutant mice were used to generate granulosa cell-specific deletion of *Cbfb* and *Runx2*. All mice were maintained on a 12 h light/dark cycle with water and food *ad libitum* at the University of Kentucky Division of Laboratory Animal Resources. For the gonadotropin-induced ovulation model, mice (25 days old) were injected with pregnant mare serum gonadotropin (PMSG, 5 IU, i.p.), followed 48 h later with human chorionic gonadotropin (hCG, 5 IU, i.p.). In our colony, the mice ovulated approximately 12 h after hCG administration^10^. To determine the stage of the estrous cycle, the vaginal fluid containing cells were obtained from adult female mice (>3 months old) daily for at least two cycles and examined microscopically.

### Genotyping

To genotype mice, ear punches were used to isolate genomic DNA using the AccuStar II Mouse Genotyping Kit (Quantabio) according to the manufacturer’s instructions. PCR was conducted with primers for *Cbfb flox*^17^, *Runx2 flox*^48^, and *Esr2* Cre^50^ listed in Supplementary Table 1, and amplified PCR products were run on 2% agarose gel for examination.

### Collection of ovaries, granulosa cells, and serum

*Cbfb*^*flox/flox*^*;Runx2*^*flox/flox*^ (wild-type) and *Cbfb*^*flox/flox*^;*Esr2*^*cre/+*^*;Runx2*^*flox/flox*^ (*gcCbfb;Runx2KO*) mice were collected at 3, 11, 16, 24, 48, or 72 h after hCG administration. Granulosa cells were isolated from ovaries collected at 11 h post-hCG via follicular puncture as described previously^9^. Serum samples were collected by cardiac puncture at the time of euthanization.

### Quantitative analysis of mRNA levels and RNA sequencing (RNA-seq) analysis

Total RNA was isolated from ovaries using a Trizol reagent (Invitrogen) and from granulosa cells using an RNeasy mini kit (QIAGEN). Levels of mRNA for genes of interest were measured by qPCR according to the method described previously^9^. Oligonucleotide primers for all genes analyzed were designed using the PRIMER3 Program and listed in Supplementary Table 1. Official full names for the genes described in the manuscript are listed in Tables as well as Supplementary Tables.

For the RNA-seq analysis, total RNA isolated from ovaries were used for library construction and then for sequencing at the DNA Services division of the Roy J. Carver Biotechnology Center at the University of Illinois. Briefly, strand-specific RNAseq libraries for eight individual samples were prepared using a TruSeq Stranded RNA Sample Prep kit (Illumina). These samples represent n = 4 independent samples per mouse line examined. The libraries were quantitated by qPCR and sequenced on one lane for 101 cycles from one end of the fragments on a NovaSeq. 6000 using a NovaSeq S2 reagent kit. Fastq files were generated and demultiplexed with the bcl2fastq v2.20 Conversion Software (https://support.illumina.com/downloads/bcl2fastq-conversion-software-v2–20.html). Adaptor sequences were trimmed from the 3’-end of the reads. Average per-base read quality scores were over 30 in all samples, indicating that those reads were high in quality. RNA-seq data were analyzed at the High Performance Computing in Biology group at the University of Illinois. Briefly, the *Mus Musculus* transcriptome and Annotation Release 106 (GRCm38.p6) from NCBI were used for quasi-mapping and count generation using Salmon (version 0.11.3)^51^. Gene-level counts were then estimated based on transcript-level counts using the biased corrected counts without an offset method from the Tximport package^52^. For the statistical analysis, the numbers of reads per gene were normalized via performing TMM (trimmed mean of M values) normalization in the edgeR package^53^ and expressed as normalized log2-based count per million values (logCPM) calculated using edgeR’s cpm() function^54^. Differential gene expression analysis for wild-type vs. *gcCbfb;Runx2KO* mice was performed using the limma-trend method on the logCPM values^55,56^. Multiple testing correction was done using the False Discovery Rate (FDR) method^57^. We defined the threshold for significant differential expression as *FDR (q)* value <0.05.

### *In Situ* localization of mRNA for *Pgr, Ptgs2, Has1, Il6, Il11, Serpine1, Adamts1, Lhcgr*, and *Prlr*

Ovaries were collected from PMSG/hCG-primed immature mice at defined times after hCG injection or from adult mice on the day of estrus. Frozen ovaries were sectioned at 10 μm and mounted on ProbeOn Plus slides (Fisher Scientific). *In situ* hybridization analysis was carried out as described previously^9,10^. Briefly, partial cDNA fragments were amplified using primers designed for mouse *Pgr, Ptgs2*, *Has1, Il6, Il11, Serpine1, Adamts1, Lhcgr*, and *Prlr* using total RNA samples isolated from ovaries at 3 or 11 h or 3 days post-hCG. The amplified PCR fragments were cloned into pCRII-TOPO Vector. Sequences of the cloned DNA were verified commercially (Eurofins Genomics). Plasmids containing partial cDNA for these genes were linearized using the appropriate restriction enzymes (e.g., EcoRV, HindIII, or BamHI). Sense and antisense riboprobes were synthesized using the corresponding linearized plasmids and labeled with Fluorescein-12-UTP. The ovarian sections hybridized with fluorescein-labeled probes were incubated with the anti-fluorescein antibody. Hybridized riboprobes were amplified using a TSA-plus fluorescein kit (Roche Applied Sciences). The sections were counterstained with propidium iodide. Specific signals were visualized with an Eclipse E800 Nikon microscope under fluorescent optics.

### Immunohistochemical analyses and apoptosis detection

Frozen sections (10 μm) were fixed using an appropriate medium (e.g., acetone, 4% paraformaldehyde, or 10% formalin solution) and incubated with primary antibodies for RUNX1 (D33G6, Cell Signaling,1:200 dilution), RUNX2 (D1L7F, Cell Signaling, 1:200 dilution), HSD3B (HPA009712, Santa Cruz Biotechnology, 1:500 dilution), CYP11A1 (D8F4F, Cell Signaling, 1:500 dilution), CLDN18 (34H14L15, Invitrogen, 1: 200 dilution), or CD31 (MEC 13.3, BD Biosciences, 1:5000 dilution) at 4 °C according to the manufacturer’s instructions^10^. After rinsing with PBS, the sections were incubated with appropriate Alexa Fluor secondary antibodies (Life Technologies), counterstained with propidium iodide, and mounted with a mounting medium (fluorogel, DABCO). Digital images were captured using an Eclipse E800 Nikon microscope, with exposure time kept constant for sections incubated with the same primary antibody.

For detection of apoptotic cells, ovarian sections were subjected to TUNEL assay using the ApopTag *in situ* apoptosis detection kit according to the manufacturer’s instructions (EMD Millipore).

### Western blot analysis

The whole cell lysate was extracted from granulosa cells collected at 11 h post-hCG using a Nuclear Extraction Kit (Active Motif) according to the manufacturer’s instructions. Cell lysates were denatured by boiling for 5 min, separated using SDS-PAGE, and transferred onto a nitrocellulose membrane. Membranes were incubated with the primary antibody against RUNX2 (SC-10758, Santa Cruz) and CBFB (33516, Abcam) overnight at 4 °C^20^. Beta-actin (Cell Signaling Technology Inc.) was used as a loading control. Blots were incubated with the respective secondary horseradish peroxidase-conjugated antibody (Santa Cruz Biotechnology) for 1 h. Peroxidase activity was visualized using the Amersham ECL Prime Western Blotting Detection Reagent (GE Healthcare).

### Immunoassay of estradiol and progesterone

Concentrations of estradiol and progesterone in serum samples were measured by an estradiol rat/mouse ELISA kit (Calbiotech) and progesterone rat/mouse ELISA kit (IBL international), respectively. Assay sensitivity for estradiol and progesterone was 3 pg/ml and 0.4 ng/ml, respectively. The intra-assay coefficient of variation for estradiol and progesterone was 9.2% and 6.2%, respectively.

### Statistical analysis

All data are presented as mean ± SEM. Statistical significance between mean values of wild-type and *gcCbfb;Runx2KO* samples was determined using an independent sample T-test in the SPSS statistics 23. Values were considered significantly different if *p*  <  0.05.

## Supplementary information

Supplementary Information.

Supplementary Information2.

## Data Availability

The datasets generated and analyzed as a part of this study are available in the NCBI Gene Expression Omnibus (GSE140922).
