# Supplementary material for: Core Binding Factors are essential for ovulation, luteinization, and female fertility in mice
Source: Sci Rep. 2020 Jun 18;10:9921. doi: 10.1038/s41598-020-64257-0 (PMC7303197; doi:10.1038/s41598-020-64257-0)
Supplement: Supplementary file 1 — Supplementary Information. [file 41598_2020_64257_MOESM1_ESM.pdf]

Core Binding Factors are essential for ovulation, luteinization, and female fertility in mice

Somang Lee-Thacker[1], Hayce Jeon[1], Yohan Choi[1], Ichiro Taniuchi[2], Takeshi Takarada[3], Yukio Yoneda[4], CheMyong Ko[5], and Misung Jo[1*]

[1]Department of Obstetrics and Gynecology, Chandler Medical Center, 800 Rose Street, University of Kentucky, Lexington, KY 40536-0298

[2]Laboratory for Transcriptional Regulation, RIKEN Center for Integrative Medical Sciences 1-7-22, Suehiro-cho, Tsurumi-ku, Yokohama, Kanagawa, 230-0045, Japan

[3]Department of Regenerative Science, Okayama University Graduate School of Medicine, Dentistry and Pharmaceutical Sciences, Okayama, 700-8558, Japan

[4]Section of Prophylactic Pharmacology, Kanazawa University, Venture Business Laboratory 402, Kakuma-machi, Kanazawa, Ishikawa, 920-1192, Japan

[5]Department of Comparative Biosciences, College of Veterinary Medicine, 2001 South Lincoln Avenue, University of Illinois at Urbana-Champaign, Urbana, Illinois 61802, USA

Supplementary Table 1) List of Primers for mRNA quantification and genotyping

Gene	DNA sequence for primers (5'-3')
Adamts1<br>(ADAM metallopeptidase with thrombospondin type 1 motif 1)	*GGACAAGCCCCAGAATCCAA*<br>*TTGCCACTGACACACCACTT*
Adamts4<br>(ADAM metallopeptidase with thrombospondin type 1 motif 4)	*CACCGAACCGACCTCTTCAA*<br>*ACCGTCAGAAAGCTTCAGGG*
Adamts5<br>(ADAM metallopeptidase with thrombospondin type 1 motif 5)	*CCAAGGCCAAATGGTGTGTC*<br>*GGTAGGCAAACTGCACTCCT*
Akr1c18<br>(aldo-keto reductase family 1, member C18)	*GATTGTGGCCCTAGCCAAGA*<br>*TAAGCAGTGATTGGAGGCGG*
Areg<br>(amphiregulin)	*AAAAGAATCCATGCACTGCC*<br>*TGTCATCCTCGCTGTGAGTC*
Ccrl2<br>(C-C motif chemokine receptor like 2)	*TCCTTCCCGACTGATACCAC*<br>*GACAAAACAGCGTCGTTTGA*
Cldn18<br>(claudin 18)	*ACACCAGATGACAGCAACTTCA*<br>*TTCATCGTCTTCTGTGCGGG*
Cyp11a1<br>(cytochrome P450 family 11 subfamily A member 1)	*GCTTAGCAGGAGGACACTGG*<br>*CTTCGATGGCAGAAGGTAGC*
Cyp17a1<br>(cytochrome P450 family 17 subfamily A member 1)	*CAATGACCGGACTCACCTCC*<br>*GAGCCTCTCCAATGCACGAT*
Edn2<br>(endothelin 2)	*CTCCTGGCTTGACAAGGAATG*<br>*GCTGTCTGTCCCGCAGTGTT*
Ereg<br>(epiregulin)	*TTCAGATGGAAGACGATCCC*<br>*CGCAACGTATTCTTTGCTCA*
Fabp4<br>(fatty acid binding protein 4)	*GTGGGAACCTGGAAGCTTGT*<br>*TGTGGTCGACTTTCCATCCC*
Fos<br>(Fos proto-oncogene, AP-1 transcription factor subunit)	*TCCTTGGAGCCAGTCAAGAG*<br>*CACAGCCTGGTGTGTTTCAC*
Has1<br>(hyaluronan synthase 1)	*TGTGTCCTGCATCAGTGGTC*<br>*GAGCGCGAGGTATACTTGGTA*
Hp<br>(haptoglobin)	*GGCTATGTGGAGCACTTGGT*<br>*TCACATTCGGGGAGTTTCTC*
Hsd3b1<br>(hydroxy-delta-5-steroid dehydrogenase, 3 beta- and steroid delta-isomerase 1)	*TTGGTGGCACTTTGCATACTT*<br>*TAGCTTTGGTGAGGGGTGTC*
Hsd17b1<br>(hydroxysteroid 17-beta dehydrogenase 1)	*GACGGAGCTCTTCTTGACTGC*<br>*TCCTGACCCGGTTTATCCGA*
Il6<br>(interleukin 6)	*ACAAGTCCGGAGAGGAGACT*<br>*ATTGCCATTGCACAACTCTTT*
Il11<br>(interleukin 11)	*CGCTGGGACATTGGGATCTT*<br>*AGGCGAGACATCAAGAGCTG*
Junb<br>(JunB proto-oncogene, AP-1 transcription factor subunit)	*CTCAGCAGTTACTCCCCAGC*<br>*CGGTCTGCGGTTCCTCTTTA*
Lhcgr<br>(luteinizing hormone/choriogonadotropin receptor)	*CGCTTTCCAAGGGATGAATA*<br>*CTGGAGGGCAGAGTTTTCAG*
Parm1<br>(prostate androgen-regulated mucin-like protein 1)	*CACCCCAAGACACTGAACCT*<br>*TATGCTGCACCTCCAAACAC*
Pgr<br>(progesterone receptor)	*CTTCCAGCTCTTTGCTGACC*<br>*AAACACCATCAGGCTCATCC*
Prlr<br>(prolactin receptor)	*TTTTGCACATGAACCCTGAA*<br>*ACCAGCAGGTGAATGTTTCC*
Ptgfr<br>(prostaglandin F receptor)	*TGTTTCCTTCTCGTGCAATG*<br>*AGATCTGATTCCACGTTGCC*
Ptgs1<br>(prostaglandin-endoperoxide synthase 1)	*CTTCTCCACGATCTGGCTTC*<br>*GAGCTGCAGGAAATAGCCAC*

Ptgs2<br>(prostaglandin-endoperoxide synthase 2)	*TGTACAAGCAGTGGCAAAGG*<br>*GCTCGGCTTCCAGTATTGAG*
Serpine1<br>(serpin family E member 1)	*GACGTTGTGGAACTGCCCTA*<br>*GACCTTTTGCAGTGCCTGTG*
Sfrp4<br>(secreted frizzled related protein 4)	*CTCAGGTATGTTGCCAGGGT*<br>*CTCAGGTATGTTGCCAGGGT*
Star<br>(steroidogenic acute regulatory protein)	*TGGAAAAGACACGGTCATCA*<br>*CAGGTCAATGTGGTGGACAG*
Timp1<br>(TIMP metallopeptidase inhibitor 1)	*CCCCAGAAATCAACGAGACCA*<br>*GTACGCCAGGGAACCAAGAA*
Wnt4<br>(Wnt family member 4)	*CTGGAGAAGTGTGGCTGTGA*<br>*GGACGTCCACAAAGGACTGT*
Wnt10b<br>(Wnt family member 10B)	*GTGGGACGCCAGGTGGTAA*<br>*AGCACCAGTGGAAACGACAG*
Runx2 flox - genotype<br>(RUNX family transcription factor 2)	*ACCAAATTAAGGGCCAGCTC*<br>*TTGAAACCATCCACAGGTGA*
Cbfb flox - genotype<br>(core-binding factor subunit beta)	*CCTCCTCATTCTAACAGGAATC*<br>*GGTTAGGAGTCATTGTGATCAC*
Esr2 cre - genotype<br>(estrogen receptor 2)	*CAGGTGCTGTTGGATGGTCTTC*<br>*CTTAGTTACTCCGGCAGCTTGAAC*<br>*AGGGGAAGTAAGGCTTGATGGTGA*

Supplementary Table 2) A list of genes differentially regulated in *gcCbfb;Runx2KO* mice

SYMBOL	GENE NAME	Fold change
*Edn2**	endothelin 2	-49.22
Cldn18	claudin 18	-16.89
*Sfrp4**	secreted frizzled-related protein 4	-15.12
Has1	hyaluronan synthase 1	-12.84
Il11	interleukin 11	-11.89
Il6	interleukin 6	-11.86
Serpine1	serine (or cysteine) peptidase inhibitor, clade E, member 1	-9.82
*Gpr141**	G protein-coupled receptor 141	-9.74
Plch2	phospholipase C, eta 2	-9.40
Akr1c18	aldo-keto reductase family 1, member C18	-9.37
Xpnpep2	X-prolyl aminopeptidase (aminopeptidase P) 2, membrane-bound	-8.67
Spp1	secreted phosphoprotein 1	-8.23
Rtl1	retrotransposon Gaglike 1	-8.04
Kl	klotho	-7.70
Rnf125	ring finger protein 125	-7.59
Slc28a2	solute carrier family 28 (sodium-coupled nucleoside transporter), member 2	-7.46
*Cemip**	cell migration inducing protein, hyaluronan binding	-7.22
*Mmel1**	membrane metallo-endopeptidase-like 1	-7.12
*Gnao1**	guanine nucleotide binding protein, alpha O	-7.03
Bdkrb2	bradykinin receptor, beta 2	-6.99
*Apol6**	apolipoprotein L 6	-6.94
Fgd3	FYVE, RhoGEF and PH domain containing 3	-6.87
*Slc12a8**	solute carrier family 12 (potassium/chloride transporters), member 8	-6.75
*Lrp8**	low density lipoprotein receptor-related protein 8, apolipoprotein e receptor	-6.61
Igsf3	immunoglobulin superfamily, member 3	-6.57
Fosb	FBJ osteosarcoma oncogene B	-6.31
Prkg2	protein kinase, cGMP-dependent, type II (PGR downstream	-6.29
Ankrd1	ankyrin repeat domain 1 (cardiac muscle)	-6.27
*Ptgfr**	prostaglandin F receptor	-6.09
Tmem71	transmembrane protein 71	-6.08
Usp43	ubiquitin specific peptidase 43	-5.76
Btg2	B cell translocation gene 2, anti-proliferative	-5.72
Ptgs2	prostaglandin-endoperoxide synthase 2	-5.66
Ptk2b	PTK2 protein tyrosine kinase 2 beta	-5.59
Tnfsf15	tumor necrosis factor (ligand) superfamily, member 15	-5.54
*Plekhf1**	pleckstrin homology domain containing, family F (with FYVE domain) member 1	-5.52
Fosl1	fos-like antigen 1	-5.50
Apol7e	apolipoprotein L 7e	-5.50
F3	coagulation factor III	-5.40
Cnga1	cyclic nucleotide gated channel alpha 1	-5.39

Ankrd55	ankyrin repeat domain 55	-5.35
Mgst2	microsomal glutathione S-transferase 2	-5.31
*Wisp2**	WNT1 inducible signaling pathway protein 2	-5.26
*Cxadr**	coxsackie virus and adenovirus receptor	-5.23
S100a6	S100 calcium binding protein A6 (calcyclin)	-5.03
Fabp4	fatty acid binding protein 4, adipocyte	-4.92
*Apln**	apelin	-4.86
*Rnf180**	ring finger protein 180	-4.85
*Hp**	haptoglobin	-4.83
Kcnj16	potassium inwardly-rectifying channel, subfamily J, member 16	-4.76
*Ptgs1**	prostaglandin-endoperoxide synthase 1	-4.74
Jakmip3	janus kinase and microtubule interacting protein 3	-4.60
*Rora**	RAR-related orphan receptor alpha	-4.58
*Entpd1**	ectonucleoside triphosphate diphosphohydrolase 1	-4.49
Zfp786	zinc finger protein 786	-4.48
Runx2	runt related transcription factor 2	-4.43
*Epdr1**	ependymin related protein 1 (zebrafish)	-4.37
Adamts1	a disintegrin-like and metallopeptidase with thrombospondin type 1 motif, 1	-4.34
Dok7	docking protein 7	-4.19
Tmem56	transmembrane protein 56	-4.19
Trib1	tribbles pseudokinase 1	-4.10
*Cabp1**	calcium binding protein 1	-4.03
Rgcc	regulator of cell cycle	-4.00
Slc16a11	solute carrier family 16 (monocarboxylic acid transporters), member 11	-4.00
Esrp2	epithelial splicing regulatory protein 2	-3.96
Cd83	CD83 antigen	-3.95
Mbp	myelin basic protein	-3.95
Slc38a3	solute carrier family 38, member 3	-3.91
Zfp469	zinc finger protein 469	-3.87
*Pfkfb3**	6-phosphofructo-2-kinase/fructose-2,6-biphosphatase 3	-3.84
Ptpn22	protein tyrosine phosphatase, non-receptor type 22 (lymphoid)	-3.77
Creg2	cellular repressor of E1A-stimulated genes 2	-3.75
*Cish**	cytokine inducible SH2-containing protein	-3.71
Stx11	syntaxin 11	-3.69
*Lhcgr**	luteinizing hormone/choriogonadotropin receptor	-3.64
Rgs16	regulator of G-protein signaling 16	-3.58
Ldhd	lactate dehydrogenase D	-3.53
Egr2	early growth response 2	-3.50
Bves	blood vessel epicardial substance	-3.50
*Dpysl4**	dihydropyrimidinase-like 4	-3.49
*Mapkapk3**	mitogen-activated protein kinase-activated protein kinase 3	-3.49
Dusp2	dual specificity phosphatase 2	-3.47
Coch	cochlin	-3.46

Pitpnc1	phosphatidylinositol transfer protein, cytoplasmic 1	-3.43
*Abcb1b**	ATP-binding cassette, sub-family B (MDR/TAP), member 1B	-3.43
Nr4a3	nuclear receptor subfamily 4, group A, member 3	-3.41
Ltbp1	latent transforming growth factor beta binding protein 1	-3.40
Atf3	activating transcription factor 3	-3.40
*Nrcam**	neuronal cell adhesion molecule	-3.34
Pdcd1	programmed cell death 1	-3.33
Ier3	immediate early response 3	-3.32
Acot4	acyl-CoA thioesterase 4	-3.31
*Sgk1**	serum/glucocorticoid regulated kinase 1	-3.31
Ngf	nerve growth factor	-3.29
Parm1	prostate androgen-regulated mucin-like protein 1	-3.24
Cyr61	cysteine rich protein 61	-3.24
Rnf152	ring finger protein 152	-3.17
Hgfac	hepatocyte growth factor activator	-3.16
Errfi1	ERBB receptor feedback inhibitor 1	-3.14
Slc39a14	solute carrier family 39 (zinc transporter), member 14	-3.14
Tbc1d30	TBC1 domain family, member 30	-3.14
Nr1h4	nuclear receptor subfamily 1, group H, member 4	-3.14
Aif1l	allograft inflammatory factor 1-like	-3.09
Efnb2	ephrin B2	-3.06
Lgr4	leucine-rich repeat-containing G protein-coupled receptor 4	-3.03
Add2	adducin 2 (beta)	-3.03
Sele	selectin, endothelial cell	-3.03
Chst15	carbohydrate sulfotransferase 15	-3.02
Avpi1	arginine vasopressin-induced 1	-3.02
Usp51	ubiquitin specific protease 51	-3.02
Vstm2a	V-set and transmembrane domain containing 2A	-3.00
Junb	jun B proto-oncogene	-2.99
Myc	myelocytomatosis oncogene	-2.99
Id2	inhibitor of DNA binding 2	-2.99
Cited4	Cbp/p300-interacting transactivator, with Glu/Asp-rich carboxy-terminal domain, 4	-2.98
Slc6a6	solute carrier family 6 (neurotransmitter transporter, taurine), member 6	-2.96
Map3k14	mitogen-activated protein kinase kinase kinase 14	-2.94
Urml	up-regulated in Myc liver	-2.94
Tnfaip3	tumor necrosis factor, alpha-induced protein 3	-2.93
*Gpx3**	glutathione peroxidase 3	-2.93
Dusp4	dual specificity phosphatase 4	-2.90
Parvb	parvin, beta	-2.89
H2-Q10	histocompatibility 2, Q region locus 10	-2.88
Ctsl	cathepsin L	-2.88
Kcnc2	potassium voltage gated channel, Shaw-related subfamily, member 2	-2.87

Fam19a2	family with sequence similarity 19, member A2	-2.85
Arhgap32	Rho GTPase activating protein 32	-2.85
Arid5a	AT rich interactive domain 5A (MRF1-like)	-2.84
Sdcbp2	syndecan binding protein (syntenin) 2	-2.83
Emb	embigin	-2.81
Psap	prosaposin	-2.80
Gimap5	GTPase, IMAP family member 5	-2.79
Adamts4	a disintegrin-like and metallopeptidase with thrombospondin type 1 motif, 4	-2.78
Irs2	insulin receptor substrate 2	-2.76
Jcad	junctional cadherin 5 associated	-2.76
Hk2	hexokinase 2	-2.75
Nipal1	NIPA-like domain containing 1	-2.74
Rdh10	retinol dehydrogenase 10 (all-trans)	-2.74
Tmem178b	transmembrane protein 178B	-2.73
Met	met proto-oncogene	-2.72
*Lin7b**	lin-7 homolog B (C. elegans)	-2.70
Sdc4	syndecan 4	-2.69
Eppk1	epiplakin 1	-2.68
Hmga1	high mobility group AT-hook 1	-2.67
Elovl7	ELOVL family member 7, elongation of long chain fatty acids (yeast)	-2.67
*Ndrg1**	N-myc downstream regulated gene 1	-2.67
Kcnab3	potassium voltage-gated channel, shaker-related subfamily, beta member 3	-2.67
Socs3	suppressor of cytokine signaling 3	-2.66
Heg1	heart development protein with EGF-like domains 1	-2.66
Pdlim5	PDZ and LIM domain 5	-2.66
*Tbc1d8**	TBC1 domain family, member 8	-2.66
Zfp398	zinc finger protein 398	-2.65
Lpgat1	lysophosphatidylglycerol acyltransferase 1	-2.64
Dusp5	dual specificity phosphatase 5	-2.63
Rnaseh2a	ribonuclease H2, large subunit	-2.62
Avil	advillin	-2.60
Apold1	apolipoprotein L domain containing 1	-2.59
Sh2b2	SH2B adaptor protein 2	-2.58
Phf20l1	PHD finger protein 20-like 1	-2.58
Rsbn1	rosbin, round spermatid basic protein 1	-2.57
Cdc73	cell division cycle 73, Paf1/RNA polymerase II complex component	-2.56
Pim1	proviral integration site 1	-2.55
Gal3st2c	galactose-3-O-sulfotransferase 2C	-2.54
Ramp1	receptor (calcitonin) activity modifying protein 1	-2.53
Ctnna2	catenin (cadherin associated protein), alpha 2	-2.52
Ankrd37	ankyrin repeat domain 37	-2.51
Arhgdib	Rho, GDP dissociation inhibitor (GDI) beta	-2.51
Csrnp1	cysteine-serine-rich nuclear protein 1	-2.50

Bcat1	branched chain aminotransferase 1, cytosolic	-2.50
Zpr1	ZPR1 zinc finger	-2.49
*Sorbs2**	sorbin and SH3 domain containing 2	-2.48
Tes	testis derived transcript	-2.48
Fos	FBJ osteosarcoma oncogene	-2.47
Ntn5	netrin 5	-2.47
Rufy4	RUN and FYVE domain containing 4	-2.47
*Ccrl2**	chemokine (C-C motif) receptor-like 2	-2.47
St3gal1	ST3 beta-galactoside alpha-2,3-sialyltransferase 1	-2.45
Cyfip2	cytoplasmic FMR1 interacting protein 2	-2.44
Pfkfb4	6-phosphofructo-2-kinase/fructose-2,6-biphosphatase 4	-2.44
Maff	v-maf musculoaponeurotic fibrosarcoma oncogene family, protein F (avian)	-2.42
Thbd	thrombomodulin	-2.39
*Timp1**	tissue inhibitor of metalloproteinase 1	-2.39
Clcf1	cardiotrophin-like cytokine factor 1	-2.38
Col9a2	collagen, type IX, alpha 2	-2.38
Stab2	stabilin 2	-2.37
Kctd11	potassium channel tetramerisation domain containing 11	-2.37
Mis18a	MIS18 kinetochore protein A	-2.37
Atg10	autophagy related 10	-2.36
Frat2	frequently rearranged in advanced T cell lymphomas 2	-2.36
*S100a4**	S100 calcium binding protein A4	-2.36
Syne3	spectrin repeat containing, nuclear envelope family member 3	-2.35
Fkbp5	FK506 binding protein 5	-2.35
Net1	neuroepithelial cell transforming gene 1	-2.35
Tpd52l1	tumor protein D52-like 1	-2.34
Rab20	RAB20, member RAS oncogene family	-2.34
Tnfrsf12a	tumor necrosis factor receptor superfamily, member 12a	-2.34
Cxcl1	chemokine (C-X-C motif) ligand 1	-2.34
Slc9a2	solute carrier family 9 (sodium/hydrogen exchanger), member 2	-2.32
Ryr2	ryanodine receptor 2, cardiac	-2.32
Lamc2	laminin, gamma 2	-2.32
*Shroom3**	shroom family member 3	-2.32
*Fam126a**	family with sequence similarity 126, member A	-2.31
Soat1	sterol O-acyltransferase 1	-2.31
Ate1	arginyltransferase 1	-2.31
Fads6	fatty acid desaturase domain family, member 6	-2.30
Sgms2	sphingomyelin synthase 2	-2.30
Calcb	calcitonin-related polypeptide, beta	-2.30
*Sdc1**	syndecan 1	-2.29
Zfp36	zinc finger protein 36	-2.29
Sphk1	sphingosine kinase 1	-2.28
Spsb4	splA/ryanodine receptor domain and SOCS box containing 4	-2.27

Jph1	junctophilin 1	-2.26
Ktn1	kinectin 1	-2.26
Ntn4	netrin 4	-2.26
*Itgb3**	integrin beta 3	-2.25
Sav1	salvador family WW domain containing 1	-2.25
Col8a1	collagen, type VIII, alpha 1	-2.24
Kcne3	potassium voltage-gated channel, Isk-related subfamily, gene 3	-2.24
Rragd	Ras-related GTP binding D	-2.23
Slc25a30	solute carrier family 25, member 30	-2.23
Hsd17b11	hydroxysteroid (17-beta) dehydrogenase 11	-2.21
Spn	sialophorin	-2.20
Map1b	microtubule-associated protein 1B	-2.19
Npas4	neuronal PAS domain protein 4	-2.19
Sspo	SCO-spondin	-2.19
Echdc2	enoyl Coenzyme A hydratase domain containing 2	-2.19
*Acsl1**	acyl-CoA synthetase long-chain family member 1	-2.18
Capn6	calpain 6	-2.18
Efs	embryonal Fyn-associated substrate	-2.18
Cyb561d1	cytochrome b-561 domain containing 1	-2.17
*Efhd1**	EF hand domain containing 1	-2.17
Gxylt2	glucoside xylosyltransferase 2	-2.16
Zc3h12a	zinc finger CCCH type containing 12A	-2.16
Casz1	castor zinc finger 1	-2.16
Hbegf	heparin-binding EGF-like growth factor	-2.12
Cebpd	CCAAT/enhancer binding protein (C/EBP), delta	-2.12
Fkbp1a	FK506 binding protein 1a	-2.12
Rem1	rad and gem related GTP binding protein 1	-2.10
Osmr	oncostatin M receptor	-2.10
Adamts5	a disintegrin-like and metallopeptidase (reprolysin type) with thrombospondin type 1 motif, 5 (aggrecanase-2)	-2.10
Ephx2	epoxide hydrolase 2, cytoplasmic	-2.10
Chsy3	chondroitin sulfate synthase 3	-2.09
Rnd1	Rho family GTPase 1	-2.09
Tle3	transducin-like enhancer of split 3	-2.08
Gpr68	G protein-coupled receptor 68	-2.08
Aplp2	amyloid beta (A4) precursor-like protein 2	-2.07
Csgalnact1	chondroitin sulfate N-acetylgalactosaminyltransferase 1	-2.07
Frat1	frequently rearranged in advanced T cell lymphomas	-2.07
Kcne4	potassium voltage-gated channel, Isk-related subfamily, gene 4	-2.07
Bhlhe40	basic helix-loop-helix family, member e40	-2.06
Hsbp1l1	heat shock factor binding protein 1-like 1	-2.06
Maml3	mastermind like transcriptional coactivator 3	-2.06
*Pcyt1a**	phosphate cytidylyltransferase 1, choline, alpha isoform	-2.05

Ostf1	osteoclast stimulating factor 1	-2.05
Gcnt2	glucosaminyl (N-acetyl) transferase 2, I-branching enzyme	-2.05
Snhg5	small nucleolar RNA host gene 5	-2.04
Mamdc2	MAM domain containing 2	-2.04
Lrp2	low density lipoprotein receptor-related protein 2	-2.03
Neat1	nuclear paraspeckle assembly transcript 1 (non-protein coding)	-2.02
Arl4d	ADP-ribosylation factor-like 4D	-2.02
Slc8b1	solute carrier family 8 (sodium/lithium/calcium exchanger), member B1	-2.02
Tspan15	tetraspanin 15	-2.02
Zfp983	zinc finger protein 983	-2.01
Vps26a	VPS26 retromer complex component A	-2.01
Basp1	brain abundant, membrane attached signal protein 1	-2.01
Rassf8	Ras association (RalGDS/AF-6) domain family (N-terminal) member 8	-2.01
Itga5	integrin alpha 5 (fibronectin receptor alpha)	-2.01
Nes	nestin	-2.00
Sbno2	strawberry notch 2	-2.00
Fndc5	fibronectin type III domain containing 5	2.02
Cd55	CD55 molecule, decay accelerating factor for complement	2.02
Vstm2b	V-set and transmembrane domain containing 2B	2.03
Pipox	pipecolic acid oxidase	2.04
Tmem255a	transmembrane protein 255A	2.04
Cx3cr1	chemokine (C-X3-C motif) receptor 1	2.08
Tcf21	transcription factor 21	2.10
Serpina3c	serine (or cysteine) peptidase inhibitor, clade A, member 3C	2.11
Rho	rhodopsin	2.14
Eda2r	ectodysplasin A2 receptor	2.15
*Plxnc1**	plexin C1	2.16
Bpifb4	BPI fold containing family B, member 4	2.17
Plac1	placental specific protein 1	2.18
Hhip	Hedgehog-interacting protein	2.22
Lair1	leukocyte-associated Ig-like receptor 1	2.22
Bpifb5	BPI fold containing family B, member 5	2.23
Hrct1	histidine rich carboxyl terminus 1	2.24
Npy1r	neuropeptide Y receptor Y1	2.26
*Prkar2b**	protein kinase, cAMP dependent regulatory, type II beta	2.27
Fam196a	family with sequence similarity 196, member A	2.28
Gnat2	guanine nucleotide binding protein, alpha transducing 2	2.29
Rasgrf2	RAS protein-specific guanine nucleotide-releasing factor 2	2.29
Smpx	small muscle protein, X-linked	2.30
Sla	src-like adaptor	2.30
Kirrel3os	kirre like nephrin family adhesion molecule 3, opposite strand	2.32
Serpina5	serine (or cysteine) peptidase inhibitor, clade A, member 5	2.33
Coasy	Coenzyme A synthase	2.34

Nap1l5	nucleosome assembly protein 1-like 5	2.34
Trank1	tetratricopeptide repeat and ankyrin repeat containing 1	2.35
*Enc1**	ectodermal-neural cortex 1	2.35
Phactr1	phosphatase and actin regulator 1	2.38
*Doc2b**	double C2, beta	2.42
Klhdc8a	kelch domain containing 8A	2.42
Pex5l	peroxisomal biogenesis factor 5-like	2.44
*Bmp3**	bone morphogenetic protein 3	2.47
Inka2	inka box actin regulator 2	2.52
Gldn	gliomedin	2.53
Pik3cg	phosphatidylinositol-4,5-bisphosphate 3-kinase catalytic subunit gamma	2.54
*Hsd3b6**	hydroxy-delta-5-steroid dehydrogenase, 3 beta- and steroid delta-isomerase 6	2.64
Plin1	perilipin 1	2.64
Cacna1a	calcium channel, voltage-dependent, P/Q type, alpha 1A subunit	2.66
*Hsd17b1**	hydroxysteroid (17-beta) dehydrogenase 1	2.72
Trpc4	transient receptor potential cation channel, subfamily C, member 4	2.75
Bcan	brevican	2.82
Klhl14	kelch-like 14	2.85
Paqr9	progestin and adipoQ receptor family member IX	2.85
Vnn3	vanin 3	2.88
Vcam1	vascular cell adhesion molecule 1	2.91
Osgin2	oxidative stress induced growth inhibitor family member 2	2.95
*Adh1**	alcohol dehydrogenase 1 (class I)	2.98
Aqp2	aquaporin 2	3.00
*Rasd1**	RAS, dexamethasone-induced 1	3.02
Phyhip	phytanoyl-CoA hydroxylase interacting protein	3.02
Nppc	natriuretic peptide type C	3.04
*Akr1b7**	aldo-keto reductase family 1, member B7	3.10
Gabra1	gamma-aminobutyric acid (GABA) A receptor, subunit alpha 1	3.12
Pnmal1	PNMA-like 1	3.14
Brinp3	bone morphogenetic protein/retinoic acid inducible neural specific 3	3.20
*Ly6d**	lymphocyte antigen 6 complex, locus D	3.31
Pkdcc	protein kinase domain containing, cytoplasmic	3.35
Ddc	dopa decarboxylase	3.39
Slfn14	schlafen 14	3.40
Egln3	egl-9 family hypoxia-inducible factor 3	3.51
Mgat4c	MGAT4 family, member C	3.61
*Slc26a7**	solute carrier family 26, member 7	3.94
Aqp5	aquaporin 5	4.06
*Inhbb**	inhibin beta-B	4.30
*Grem1**	gremlin 1, DAN family BMP antagonist	4.46
Ano3	anoctamin 3	4.65

Cyp4f15	cytochrome P450, family 4, subfamily f, polypeptide 15	4.75
Cyp17a1	cytochrome P450, family 17, subfamily a, polypeptide 1	6.16
*Cyp19a1**	cytochrome P450, family 19, subfamily a, polypeptide 1	6.47
Gpr83	G protein-coupled receptor 83	9.71

The differentially regulated genes meeting the criteria of the fold change of 2 or greater and average expression value of 0.5 or greater are listed (342 genes). Fold changes were calculated by comparing the average values for mutant mouse samples vs. control mouse samples. Lower expression in mutant mouse samples is given as -2 rather than 0.5.
Of note, the differential regulation of *Cyp19a1* mRNA in the KO mice was assessed to verify RNA-seq data. However, both RT-qPCR and *in situ* hybridization analyses showed no significant difference in the levels as well as localization of *Cyp19a1* mRNA. Therefore, we have excluded this gene from the selected list (Table 2).
