# Supplementary material for: Core Binding Factors are essential for ovulation, luteinization, and female fertility in mice
Source: Sci Rep. 2020 Jun 18;10:9921. doi: 10.1038/s41598-020-64257-0 (PMC7303197; doi:10.1038/s41598-020-64257-0)
Supplement: Supplementary file 2 — Supplementary Information2. [file 41598_2020_64257_MOESM2_ESM.pdf]

Core Binding Factors are essential for ovulation, luteinization, and female fertility in mice

Somang Lee-Thacker[1], Hayce Jeon[1], Yohan Choi[1], Ichiro Taniuchi[2], Takeshi Takarada[3], Yukio Yoneda[4], CheMyong Ko[5], and Misung Jo[1*]

[1]Department of Obstetrics and Gynecology, Chandler Medical Center, 800 Rose Street, University of Kentucky, Lexington, KY 40536-0298

[2]Laboratory for Transcriptional Regulation, RIKEN Center for Integrative Medical Sciences 1-7-22, Suehiro-cho, Tsurumi-ku, Yokohama, Kanagawa, 230-0045, Japan

[3]Department of Regenerative Science, Okayama University Graduate School of Medicine, Dentistry and Pharmaceutical Sciences, Okayama, 700-8558, Japan

[4]Section of Prophylactic Pharmacology, Kanazawa University, Venture Business Laboratory 402, Kakuma-machi, Kanazawa, Ishikawa, 920-1192, Japan

[5]Department of Comparative Biosciences, College of Veterinary Medicine, 2001 South Lincoln Avenue, University of Illinois at Urbana-Champaign, Urbana, Illinois 61802, USA

A)

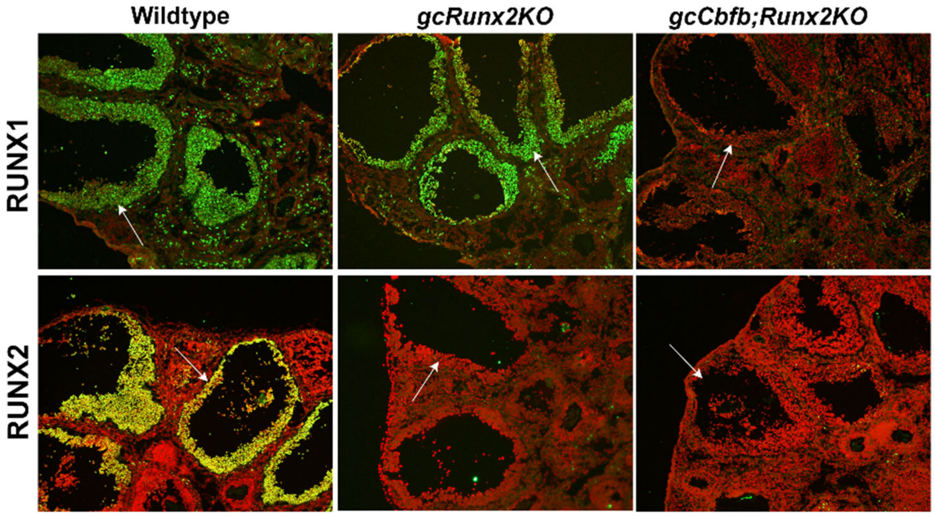

B)

Genotype	n	Fertility	# of pups/litter	# of litters
**Wild-type**	9	100%	8.7 ± 0.4	2.3 ± 0.16 (for 3 months)
gcRunx2KO	6	50% (3*/6)	4.3 ± 1.2 by *	1 by * (for 2 months)
gcCbfb;Runx2KO	9	0%	0	0 (for 3 months)

C)

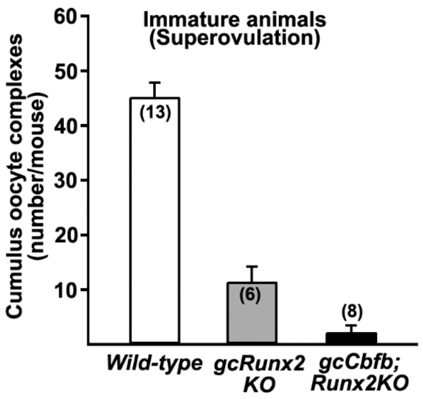

D)

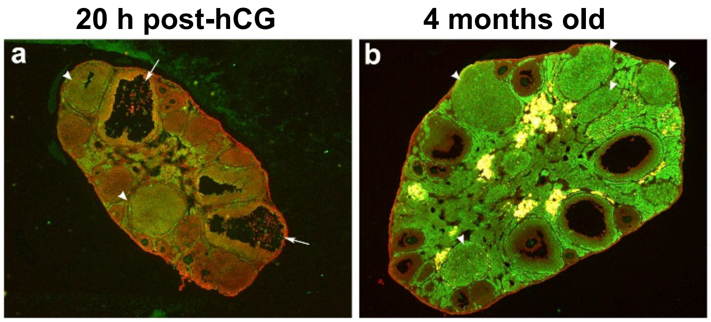

Supplementary Fig. 1: Characterization of ovarian and fertility phenotype of $Cbfb^{flox/+}$;$Esr2^{cre/+}$;$Runx2^{flox/flox}$ mice (referred to *gcRunx2KO*).

A) Ovaries collected at 11 or 12 h post-hCG administration was used to assess the expression of *Runx1* and *Runx2* in periovulatory follicles in the ovary of wild-type ($Cbfb^{flox/}$*flox*;$Runx2^{flox/flox}$), *gcRUNX2KO*, and *gcCbfb;Runx2KO* ($Cbfb^{flox/flox}$;$Esr2^{cre/+}$;$Runx2^{flox/flox}$). In *gcRunx2KO* mice, RUNX2 was not detected, while strong positive staining for RUNX1 was localized to granulosa cells of periovulatory follicles. In *gcCbfb;Runx2KO* mice, both RUNX1 and RUNX2 were detected in periovulatory follicles at 11 h post-hCG. Green/yellow staining presents positive staining for RUNX1 and RUNX2. The sections were counterstained with propidium iodide (red staining). Arrows point to periovulatory follicles.

B) Fertility was assessed by mating female mice (~2 months old) with fertile males. Six female *gcRunx2KO* mice were mated with fertile males for 2 -3 months; 3 *gcRunx2KO* mice had pups (2, 6, 4 pups for each), while other 3 mice did not show any sign of pregnancy, indicating these mice were subfertile. * denotes the mice that had pups.

C) Ovulation rates were evaluated using immature mice (~25 days old). These animals were administered with PMSG/hCG to induce superovulation and euthanized between 16 and 26 hours after hCG injection, and cumulus complexes were collected from oviducts and counted. The number inside/outside each bar represents the sample size of mice used for each experiment.

D) The images of the ovary of *gcRunx2KO* mice collected at 20 h after hCG administration from immature mice (a) and unstimulated adult mice (b). HSD3B staining (yellow/green) was used to mark periovulatory follicles and CL. Arrows point to unruptured follicles with entrapped expanded cumulus cells, suggesting the failure of ovulation in several ovulatory follicles, but not all. Arrowheads point to corpora lutea, indicating that large antral follicles eventually transform into the CL. The sections were counterstained with propidium iodide (red staining).

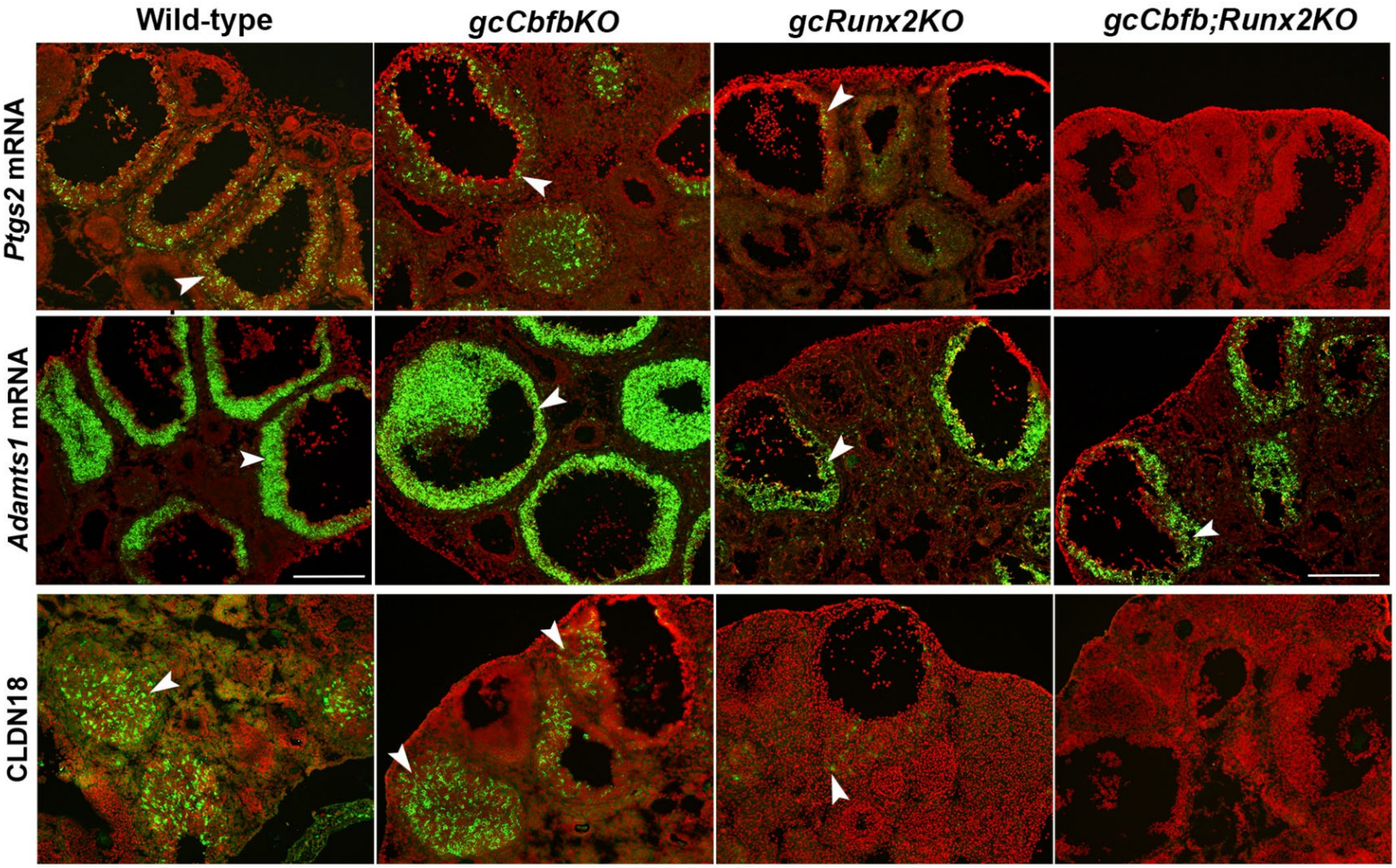

Supplementary Fig. 2) The expression of *Ptgs2*, *Adamts1*, and *Cldn18* was reduced in *gcRunx2KO* (*Cbfb*$^{flox/+}$;*Esr2*$^{cre/+}$;*Runx2*$^{flox/fox}$) and *gcCbfb;Runx2KO* (*Cbfb*$^{flox/flox}$;*Esr2*$^{cre/+}$;*Runx2*$^{flox/fox}$) mice. Ovaries were collected from wild-type (*Cbfb*$^{flox/flox}$;*Runx2*$^{flox/fox}$), *gcCbfbKO* (*Cbfb*$^{flox/flox}$;*Esr2*$^{cre/+}$), *gcRunx2KO* and *gcCbfb;Runx2KO* mice. *In situ* hybridization analysis was used to localize *Ptgs2* and *Adamts1* mRNA in ovaries collected during the late ovulatory period between 11 or 12 h after hCG administration. Immunohistochemical analysis was used to detect CLDN18 protein in ovaries collected during the post-ovulatory period (~24 h post-hCG). Arrows point to the cells positively stained for *Ptgs2* mRNA, *Adamts1* mRNA, and CLDN18. Scale bars, 250 μm for all the image

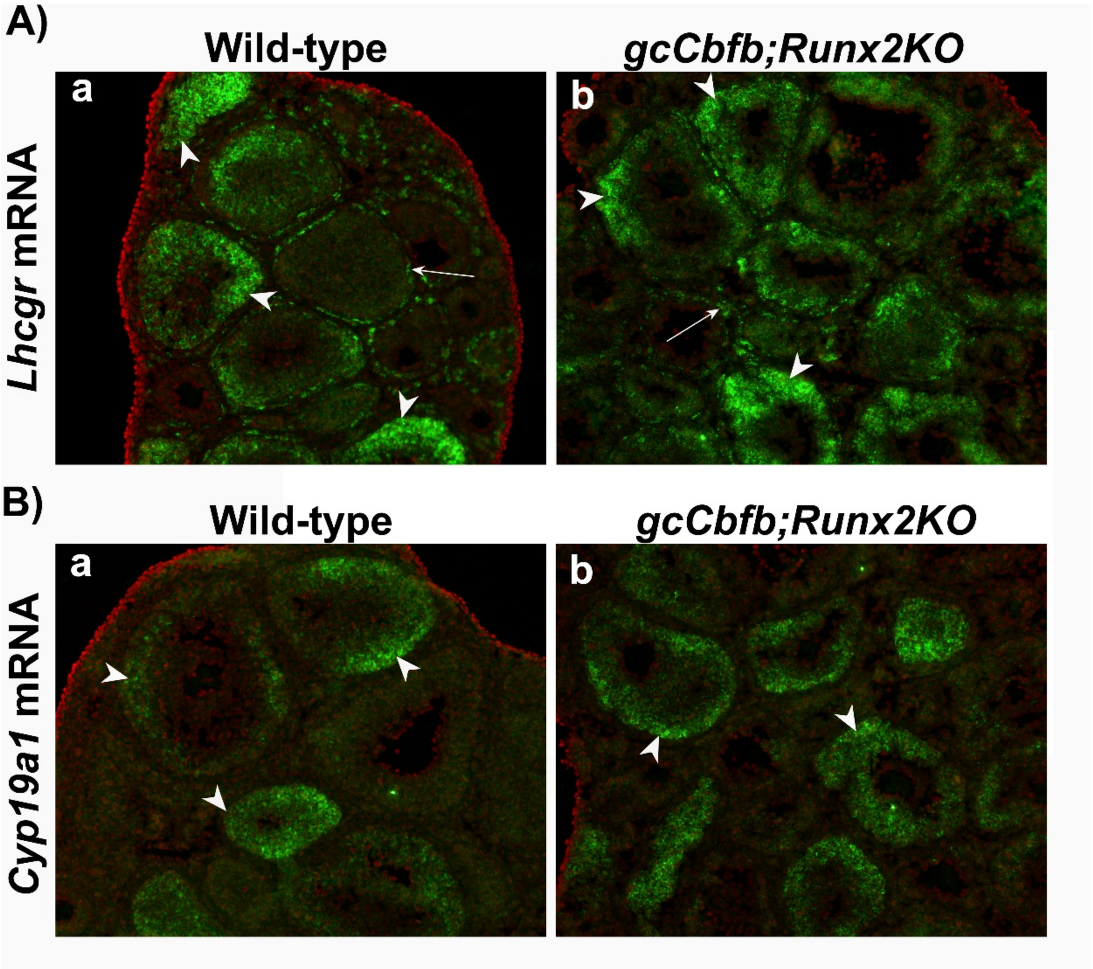

Supplementary Fig. 3) The assessment of *Lhcgr* and *Cyp19a1* expression in the ovary of *gcCbfb;Runx2KO* (*Cbfb$^{flox/flox}$;Esr2$^{cre/+}$;Runx2$^{flox/flox}$*) mice during follicular development before hCG administration. The ovaries (n=2 animals/genotype) were collected at 24 h after PMSG administration. The expression of mRNA for *Lhcgr* and *Cyp19a1* was evaluated by *In situ* hybridization analysis as described in the Material and Method section in the main manuscript. A) *Lhcgr* mRNA (green fluorescent staining) was localized to granulosa (arrowheads) and theca cells (arrows) in both wild-type and *gcCbfb;Runx2KO* mice. B) *Cyp19a1* mRNA (green fluorescent staining) was localized to granulosa cells (arrowheads) of both wild-type and *gcCbfb;Runx2KO* mice. The tissue sections were lightly counterstained with propidium iodide (red staining). Magnification, 100x. These data indicated that *Lhcgr* and *Cyp19a1* mRNA were expressed in granulosa cells before hCG administration in *gcCbfb;Runx2KO* mice similar to those observed in the ovary of wild-type mice.
